# Novel Thioredoxin reductase 1 inhibitor BS1801 relieves treatment resistance and triggers endoplasmic reticulum stress by elevating reactive oxygen species in glioma

**DOI:** 10.1016/j.redox.2025.103827

**Published:** 2025-08-29

**Authors:** Yuanhao Chang, Jin Chang, Jiayu Ji, Jing Sun, Huimin Hu, Bo Pang, Qi Zhang, Hanwei Yin, Huihui Zeng, Tao Jiang, Guanzhang Li, Fan Zeng

**Affiliations:** a Department of Neurosurgery, Beijing Tiantan Hospital, Capital Medical University, No.119 South 4th Ring Road West, Beijing, China; b Department of Molecular Neuropathology, Beijing Neurosurgical Institute, Capital Medical University, No.119 South 4th Ring Road West, Beijing, China; c China National Clinical Research Center for Neurological Diseases, Beijing, China; d Chinese Glioma Genome Atlas Network (CGGA) and Asian Glioma Genome Atlas Network (AGGA), Beijing, China; e School of Bioengineering, Tianjin University of Science and Technology, Tianjin, China; f China Rehabilitation Science Institute, China Rehabilitation Research Center, School of Rehabilitation, Capital Medical University, Beijing, China; g State Key Laboratory of Natural and Biomimetic Drugs, No. 38, Xueyuan Road, Beijing, China; h Department of Chemical Biology, School of Pharmaceutical Sciences, Peking University, No. 38, Xueyuan Road, Beijing, China; i Department of Ultrastructural Pathology, Beijing Neurosurgical Institute, Capital Medical University, No.119 South 4th Ring Road West, Beijing, China; j Keaise Center for Clinical Laboratory, No. 666, Gaoxin Road, Wuhan, China; k Beijing Engineering Research Center of Targeted Drugs and Cell Therapy for CNS Tumors, Beijing, China

**Keywords:** Glioma, Temozolomide resistance, Thioredoxin reductase inhibitor, Reactive oxygen species, Endoplasmic reticulum stress

## Abstract

Glioma patients will inevitably develop resistance to temozolomide (TMZ) leading to tumor recurrence. By comparing genomic differences between primary and recurrent glioma patients, Thioredoxin reductase 1 (TrxR1) was identified as a crucial role in TMZ resistance. Glioma cells elevate the expression level of *TXNRD1* to against TMZ-induced reactive oxygen species (ROS), thereby conferring TMZ resistance. BS1801 is a novel small-molecular targeted drug that binds to Cys497/Sec498 activity site of TrxR1, competitively inhibiting its activity. The results showed that glioblastoma cells were time- and dose-dependently inhibited by BS1801 treatment. Additionally, BS1801 treatment elevated ROS levels, resulting in glioblastoma cell cycle arrest, endoplasmic reticulum (ER) stress, mitochondrial dysfunction and apoptosis. Meanwhile, BS1801 synergized with TMZ to significantly inhibit glioblastoma cell proliferation, induce DNA damage and trigger mitochondrial depolarization. The modified BS1801-nano combined with TMZ treatment significantly prolonged the overall survival of intracranial orthotopic glioma mice models. Finally, a predictive model for BS1801 treatment sensitivity was established using patient-derived GBM organoids. In summary, BS1801 treatment can elevate ROS levels, induce glioblastoma cell apoptosis and activate ER stress, thereby relieving TMZ resistance. BS1801 exhibits potent glioma inhibitory effects and potential clinical application.

## Highlights

• Large-scale sequencing data identifies *TXNRD1* is a key factor of temozolomide resistance in recurrent glioma patients.
• Novel TrxR1 inhibitor BS1801 triggers ER stress and mitochondrial damage to induce glioma cells apoptosis.
• The establishment of glioblastoma patients-derived organoids may screen out BS1801 sensitive patients.
• Nanocrystal BS1801 elevates the permeability of Blood-Brain-Barrier.

## Graphical abstract

New drugs are urgently needed for recurrent glioma patients with TMZ resistance. Novel targeted drug BS1801 interacts with TrxR1 at Cys497/Sec498 activity site to inhibit enzyme activity, resulting in elevating ROS levels. High concentrations of ROS trigger ER stress, mitochondrial dysfunction and DNA damage to induce cell apoptosis, thereby relieving TMZ resistance. BS1801 exhibits potent glioma inhibitory effects and potential clinical application.

**Figure:**
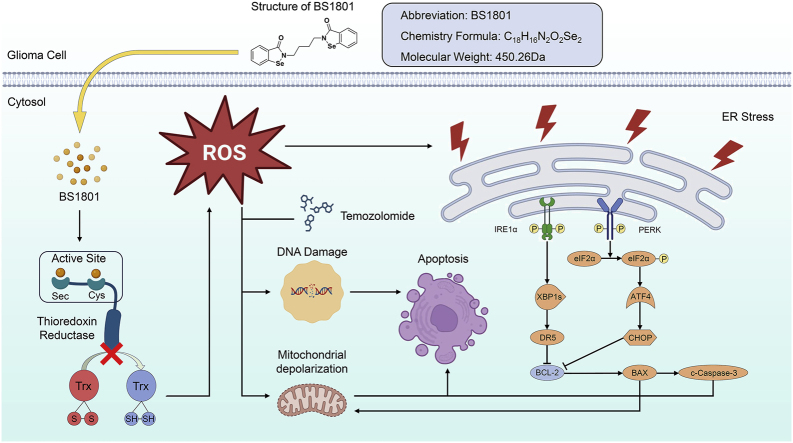

## 1 Introduction

Glioma is the most common primary malignant brain tumors in adults, accounting for more than 80 % of all intracranial malignancies [[1], [2], [3], [4]]. Among them, WHO grade IV glioblastoma multiforme (GBM) exhibits the highest incidence rate [5]. Current clinical treatment for primary GBM patients involves maximal surgical resection combined with normative Stupp protocol [6]. Stupp is a regimen of radiotherapy combined with continuous daily TMZ, followed by 6 cycles of adjuvant TMZ therapy. Though comprehensive treatment, the prognosis of GBM patients remains poor, with a mean median survival of only 14.6 months [7,8]. In the past two decades, no new drugs designed for GBM have been approved by FDA, and TMZ remains the only first-line chemotherapeutic agent [2,9,10]. However, GBM patients will soon develop resistance after TMZ treatment [11]. Hence, elucidating the potential causes of TMZ resistance and searching for new therapeutic drugs will be feasible and urgent.

As an alkylating agent, TMZ primarily exerts cytotoxicity through DNA damage. To versus this lesion, glioma cells activate DNA repair pathways to stabilize genome, resulting in TMZ resistance [12]. Recent studies have reported that O^6^-methylguanine-DNA methyltransferase (MGMT) induces TMZ resistance in GBM via removal of TMZ-induced alkylation from different nucleotides [11,13,14]. Meanwhile, the methylation status of MGMT promoters have been identified as a biomarker to predict TMZ resistance in clinical [15]. However, accumulating evidence suggests that the development of TMZ resistance is a rather complicate process [[16], [17], [18]]. Therefore, MGMT is not the only factor.

During tumorigenesis, there are biological processes including maintaining proliferation signals, evading growth inhibition, resisting cell death, inducing angiogenesis, reprogramming metabolism, and evading immune destruction [[19], [20], [21]]. Redox signaling is involved in almost all cancer-related biological processes [22]. Studies have shown that alkylating agents, such as cyclophosphamide and lomustine, can induce oxidative stress in cancer cells and subsequently activate the antioxidant defense system to develop resistance [23,24]. Most anti-tumor drugs kill their target cells at least partially by producing large amounts of intracellular ROS [25]. ROS serves as a crucial second messengers in intracellular signaling pathways and plays a conflicting dual role. Several studies reported that moderate ROS levels induce and maintain the oncogenic phenotypes in cancer cells [26,27], whereas excessive ROS may damage proteins and DNA, resulting in cancer cell apoptosis [28]. Therefore, the role of ROS in glioma cells need to be further investigated.

In this study, we characterized TMZ resistant patients using RNA-seq data from recurrent glioma tumors and corresponding clinical data. We identified that low levels of ROS may contribute to TMZ resistance and *TXNRD1* was the most significantly upregulated gene during TMZ resistance. Based on these results, we designed and evaluated the efficacy of TrxR1-targeted drug BS1801. By applying cell line experiments, animal models and GBM organoids, we ascertained the mechanism and synergistic effect of BS1801 in inhibiting glioma proliferation. Our study provides a novel therapeutic strategy for TMZ resistant glioma patients and exhibited clinical application prospects.

## 2 Materials and methods

### 2.1 Ethics approval and data collection

Tissue samples were collected from glioma patients undergoing surgery at Capital Medical University Affiliated Beijing Tiantan Hospital according to the protocol approved by the Ethics Committee (No. KY2013-017-01). Transcriptome sequencing data were uploaded to our public database website (https://www.cgga.org.cn). CGGA database and CGGA (2019) database contained 325 and 693 patient samples, respectively. Other clinical treatment information and pathological details were shown in Supplementary Table S1–2. GBM patients-derived cells (BNI-19-1 and BNI-20-1) and GBM organoids were obtained from isolated GBM tissues according to the protocol approved by the Ethics Committee of Capital Medical University Affiliated Beijing Tiantan Hospital (No. KY2020-093-02). Written informed consent was obtained from all patients for the use of associated data and samples in this study. The animal study protocol in this study was approved by Beijing Neurosurgical Institute according to National Institutes of Health guidelines (No. 202002002). All animal procedures followed the guidelines of Institutional Animal Care and Use Committee.

### 2.2 Chemicals

BS1801 (MW: 450.26 g/mol; molecular formula: C_18_H_16_N_2_O_2_Se_2_; chemical name: 2,2'-(butane-1,4-diyl)bis(benzo[d] [1,2]selenazol-3(2H)-one); CAS No. 857375-83-8; properties: light yellow solid; melting point: >300 °C; solubility: soluble in DMF and DMSO, insoluble in water, slightly soluble in dichloromethane; pKa (Predicted) Value: 1.70 ± 0.20; miLogP: 3.51) was synthesized in the State Key Laboratory of Natural and Biomimetic Drugs, Peking University. The structure of BS1801 was shown in Fig. S4A. Temozolomide (HY-17364), MKC8866 (HY-104040), GSK2606414 (HY-18072) and Acetylcysteine (HY-B0215) were purchased from MedChemExpress. Sodium carboxymethylcellulose was purchased from RHAWN (R149528). TRIzol was purchased from Thermo Fisher (15596026).

**Fig. 1:**
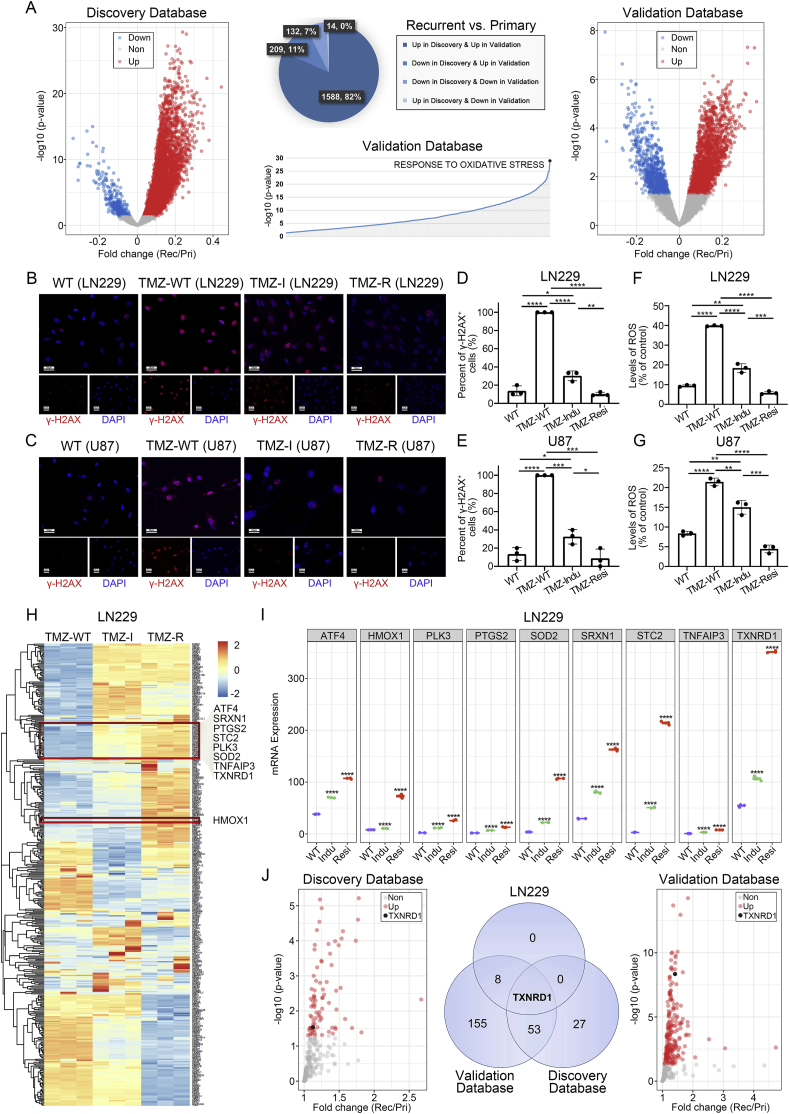
**Association of oxidative stress status with TMZ resistance.** (A) Volcano maps showed the changes in biological functions in recurrent glioma patients compared with primary glioma patients. Red dots indicated elevated biological functions in recurrent patients. Blue dots indicated decreased biological functions in recurrent patients. Grey dots indicated non-significant biological functions. Black dot indicated significantly enhanced “response to oxidative stress” function in validation database. (B–C) IF staining of γ-H2AX in different status of LN229 and U87 cell lines. Red fluorescence represented γ-H2AX. Cell nuclei were stained with DAPI. Scale bar was 20 μm. (D–E) Bar plot showed the percent of γ-H2AX positive cells in LN229 and U87 IF images. (F–G) Flow cytometry detected ROS levels in different status of LN229 and U87 cell lines. (H) Heatmap showed differential gene expressions in three statuses of LN229 cells. Oxidative stress related genes were highlighted in red box. (I) Scatter diagram showed the expression levels of nine oxidative stress related genes were elevated in TMZ resistance progress. (J) Venn diagram showed *TXNRD1* was the significantly elevated gene among the three RNA-sequencing results. WT: non-treatment cells; TMZ-WT: TMZ treatment for 72 h; TMZ-I: iterative pulse exposure of TMZ; TMZ-R: TMZ-resistant cells. N = 3 for each group. ∗, ∗∗, ∗∗∗ and ∗∗∗∗ indicated that p < 0.05, p < 0.01, p < 0.001 and p < 0.0001, respectively.

**Fig. 2:**
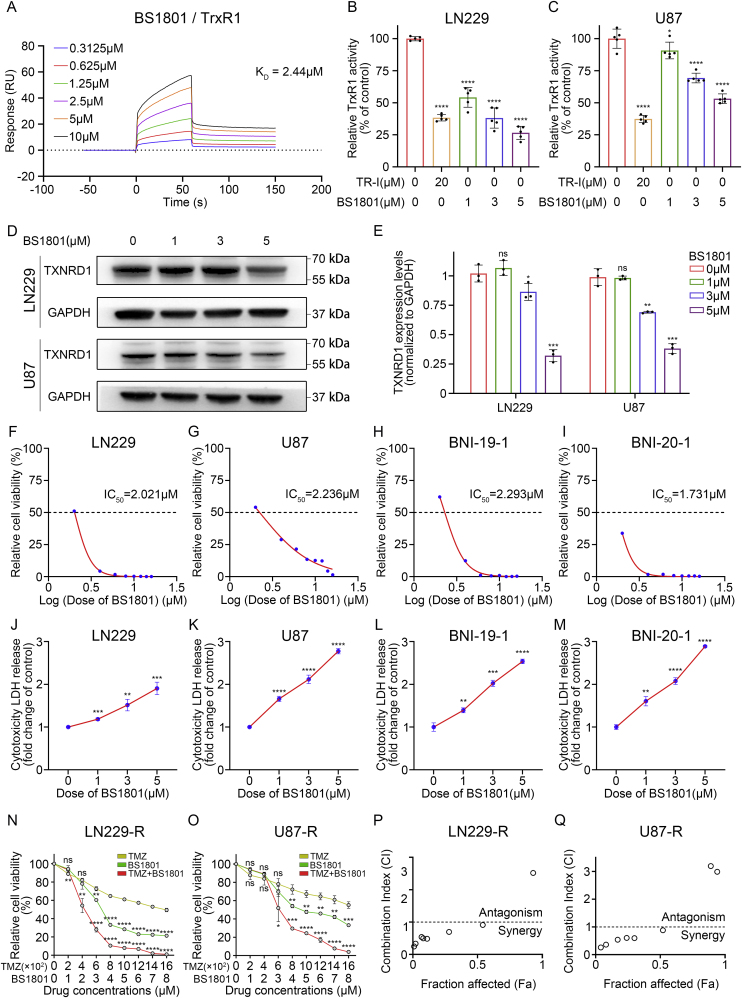
**BS1801 inhibited TrxR1 activity and glioma cell growth.** (A) The binding affinity of BS1801 with recombination human TrxR1 protein were determined by using SPR assay. K_D_: dissociation constant. (B–C) DTNB assay revealed that the TrxR1 inhibition rates after BS1801 treatment on LN229 and U87 cell lines for 24 h. TR-I was a strong TrxR inhibitor and served as a positive control. N = 5 for each group. (D–E) Immunoblots for TXNRD1 in LN229 and U87 cell lines after BS1801 treatment for 24 h. GAPDH was probed as loading control. (F–I) CCK8 test detected the cell viability after different concentrations of BS1801 treatment in LN229, U87, BNI-19-1 and BNI-20-1 cell lines for 24 h. (J–M) Cytotoxicity LDH release assay measured LDH contents after different concentrations of BS1801 treatment in LN229, U87, BNI-19-1 and BNI-20-1 cell lines for 24 h. (N–Q) CCK8 test detected the cell viability after different concentrations of BS1801 and TMZ treatment in TMZ resistant LN229 and U87 cell lines. TMZ: BS1801 = 200: 1. Fa-CI curve reflected synergy or antagonism effect of BS1801 and TMZ at different concentrations in TMZ resistant cell lines. N = 3 for each group. ns, ∗, ∗∗, ∗∗∗ and ∗∗∗∗ indicated that no significance, p < 0.05, p < 0.01, p < 0.001 and p < 0.0001, respectively.

### 2.3 Preparation of BS1801-nano

Wet-milling technology was used to prepare BS1801 nanocrystals. Firstly, PVPk-30 and SDS were completely dissolved in pure water to form aqueous solution. Then BS1801 powder was dispersed in solution. The mixture was stirred with a magnetic stirrer for 5 min at 1000 rpm, followed by stirring with an emulsifying homogenizer at 3000 rpm for 5 min to produce a crude drug suspension. These crude suspensions were further grinded with zirconium oxide beads (0.3 mm diameter) in an AK71M-2WKF wet grinder at 3000 rpm for an hour. Finally, the particle size and zeta potential of BS1801-nano were measured by dynamic light scattering analysis (DLS) using a Malvern Zetasizer Nano ZS at 25 °C.

### 2.4 Cell lines and cell culture

**Supplementary Fig. S1:**
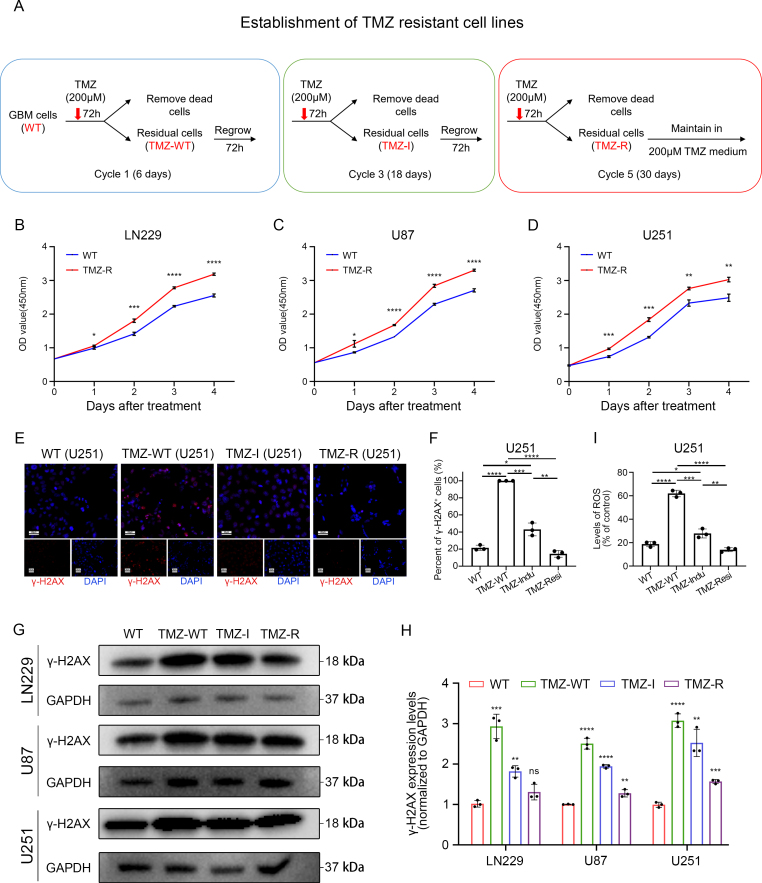
Association of oxidative stress status with TMZ resistance. (A) The workflow of establishment of TMZ resistant cell lines. (B-D) CCK8 test detected the cell viability of wildtype and TMZ resistant glioma cell lines with 200 μM TMZ treatment at different times. (E) IF staining of γ-H2AX in different status of U251 cell line. Red fluorescence represented γ-H2AX. Cell nuclei were stained with DAPI. Scale bar was 20 μm. (F) Bar plot showed the percent of γ-H2AX positive cells in U251 IF images. (G-H) Immunoblots for γ-H2AX in different status of LN229, U87 and U251 cell lines. GAPDH was probed as loading control. (I) Flow cytometry detected ROS levels in different status of U251 cell line. WT: non-treatment cells; TMZ-WT: TMZ treatment for 72 h; TMZ-I: iterative pulse exposure of TMZ; TMZ-R: TMZ-resistant cells. N = 3 for each group. ns, ∗, ∗∗, ∗∗∗ and ∗∗∗∗ indicated that no significance, p < 0.05, p < 0.01, p < 0.001 and p < 0.0001, respectively.

LN229, U87 and U251 cells were purchased from National Infrastructure of Cell Line Resource and cultured in DMEM medium (Gibco) with 10 % FBS (Gibco) and 1 % penicillin/streptomycin (Gibco). TMZ-resistant cells were established by treating parental glioma cells with iterative pulse exposure to TMZ. When the cells reached 70 %–80 % confluence, 200 μM TMZ was added. After 3 days of TMZ exposure, TMZ-WT cells were obtained and the medium was replaced with fresh TMZ-free medium until the living cells recovered. When the cells reached an equal confluence, TMZ was added again and the first cycle was completed. After three cycles, TMZ-I cells were obtained and revealed potential TMZ resistance. Finally, after five cycles, TMZ-resistant (TMZ-R) cells were successfully established and maintained in 200 μM TMZ (Fig. S1A). Patients-derived BNI-19-1 and BNI-20-1 cells were cultured in serum-free DMEM/F12 medium (Gibco) supplemented with 2 % B27 (Gibco), 20 ng/ml basic fibroblast growth factor (bFGF, PeproTech) and 20 ng/ml epidermal growth factor (EGF, PeproTech). Growth factors (bFGF and EGF) were added twice a week. All cell lines were cultured in the incubator containing 5 % CO_2_ at 37 °C.

### 2.5 Bioinformatics analysis

Gene set variation analysis, Pearson correlation analysis, Prognosis analysis and Nomogram analysis were performed with the “GSVA”, “corrplot”, “survival” and “rms” packages in R software (version 4.2.0), respectively. The lists of biological function were obtained from the Gene Set Enrichment Analysis database (https://www.gsea-msigdb.org/gsea/msigdb/index.jsp). Patient survival distribution and significance were evaluated by Kaplan-Meier plot and log-rank test. The susceptibility of BS1801 in glioma patients could be exactly predicted by total points, the sum points of every prognostic factor. Receiver operator characteristic curves validated the accuracy of BS1801 susceptibility prediction. Other details were described in our previous study [29].

### 2.6 RNA sequencing

Total RNA of GBM cell lines and GBM organoids were extracted using the TRIzol reagent according to the manufacturer's protocol. RNA purity and quantification were evaluated using the NanoDrop 2000 spectrophotometer (Thermo Fisher). The libraries were constructed using VAHTS Universal V6 RNA-seq Library Prep Kit and the transcriptome sequencing data was generated by the llumina Novaseq 6000 platform. The student's t-test was performed to identify differentially expressed genes (p < 0.05). Heatmap and volcano plot were used to visualize the differential genes.

### 2.7 Gene ontology (GO) and Kyoto encyclopedia of genes and genomes (KEGG) analyses

Pearson correlation analysis was performed to screen out *TXNRD1* related genes (R > 0.6, p < 0.0001) in discovery and validation databases. The biological functions and signaling pathways related to *TXNRD1* were explored by GO and KEGG analyses using the DAVID bioinformatics resource (version 6.7) [30], an online functional annotation tool (https://david.ncifcrf.gov/). GO and KEGG results of the most correlated genes were visualized by bubble plot and interaction diagram, respectively.

### 2.8 Immunofluorescence (IF) staining

3 × 10^5^ GBM cells were seeded into confocal plates (NEST) and treated with different chemicals. After incubating for 24 h, 4 % paraformaldehyde (Solarbio) solution was added to fix cells for 10 min. Subsequently, 0.5 % Triton X-100 (Solarbio) was added to the plates for 10 min. After washing, 5 % BSA (Solarbio) was added and incubated for 1 h at room temperature. Then cells were incubated with primary antibodies overnight at 4 °C. After washing with PBS, the cells were incubated with secondary antibodies for 1 h at room temperature. Cells were washed again and treated with DAPI (Invitrogen). Finally, the photos were taken with confocal microscopy.

### 2.9 TrxR1 enzyme activity measurement

GBM cells were treated with different concentrations of BS1801 for 24 h. After washing, cells were lysed by lysis buffer (CelLytic™ M) containing 1 % PMSF (Solarbio) to extract protein. Mammalian thioredoxin reductase activity was determined with Thioredoxin Reductase Assay Kit (Sigma-Aldrich) using DTNB as the substrate. 20 μM TR-I treatment was used as positive control.

### 2.10 Cell viability

Cell Counting Kit-8 (CCK8) assay was used to evaluate cell proliferation and cytotoxicity. Cells at a density of 5 × 10^3^ cells/well were seeded in 96-well plates and incubated overnight under 5 % CO_2_ at 37 °C, following by exposure to a series of concentrations of TMZ or BS1801. Each group had five biological repeats. After dosing for 24 h, fresh medium (100 μL) supplemented with 10 μL CCK8 solution was added to each well. After incubating in the dark for 2 h at 37 °C, the optical density at 450 nm of each well were measured. Cell viability was calculated according to the previous study [31].

### 2.11 Real-time cell proliferation analysis

In brief, background impedance was measured in cell culture medium per well. 50 μL DMEM medium was placed in the E-plate 8 and incubated at 37 °C with 5 % CO_2_ for 24 h. Then 100 μL GBM cells suspension (5000 per well) with DMEM medium were added into the plate and maintained the plate at room temperature for 30 min. After 12 h incubation, the medium was changed with fresh medium containing different concentrations of BS1801 and TMZ, and the cell index was recorded every 5 min for 72 h. Each independent experiment was performed in triplicate. The interval slope was calculated automatically by the RTCA software to evaluate the rate of cell index change.

### 2.12 Cytotoxicity lactic dehydrogenase (LDH) measurement

Cytotoxicity LDH Assay Kit-WST (Dojindo) was used to measure the LDH levels in supernatant after BS1801 treatment. GBM cells at a density of 5 × 10^3^ cells/well were seeded in 96-well plates and incubated overnight. Subsequently, the cells were exposed to a series of BS1801 concentrations. After 24 h treatment, the 96-well plate was centrifuged at 1000 r/min for 5 min. Then the supernatants were collected to conduct LDH level measurement.

### 2.13 Flow cytometry

GBM cells were pretreated with different concentrations of BS1801 (0, 1, 3, 5 μM) or NAC (5 mM) for 24 h and then collected in tubes. For ROS level measurement, cells were stained with DCFDA (Abcam) for 30 min and detected at FL-1 channel. For cell cycle analysis, Cell Cycle Analysis Kit (BD Biosciences) was used for propidium iodide (PI) staining according to the manufacturer's protocol. The fluorescence signals were collected from FL-3 channel. For apoptosis assay, Annexin-V/PI Apoptosis Analysis Kit (Invitrogen) was used for cells staining. The Annexin-V and PI signals were collected from FL-1 and FL-3 channels, respectively. Each sample was analyzed in triplicate. Data were acquired by the flow cytometer (BD, Accuri C6) and analyzed with Flowjo (V10) software.

### 2.14 Glutathione (GSH) and glutathione disulfide (GSSG) levels measurement

GBM cells were seeded to 96-well plate at density of 5 × 10^3^/well. After incubating for 24 h, cells were exposed to different concentrations of BS180 for 24 h. GSH/GSSG-Glo™ Assay kit (Promega) was used to measure GSH and GSSG levels. Total Glutathione Lysis Reagent and Oxidized Glutathione Lysis Reagent were used to measure the levels of GSH + GSSG and GSSG, respectively. Fluorescence values of two groups were measured and the GSH/GSSG ratio was calculated as follows: GSH/GSSG = (GSH + GSSG – GSSG × 2)/GSSG.

### 2.15 Tunel assay

Apoptosis in tumor tissue was analyzed by applying terminal deoxynucleotidyl transferase-mediated dUTP nick end-labeling (TUNEL) staining according to the manufacturer's instructions. TUNEL assay results were visualized by fluorescence microscopy, and TUNEL-positive nuclei were stained green in fluorescence images. The apoptotic rate was calculated as the proportion of TUNEL-positive cells per field.

### 2.16 Transmission electron microscopy (TEM)

LN229 cells were treated with different concentrations of BS1801 (0, 1, 3, 5 μM) for 24 h in 6-well plates (1 × 10^6^ cells/well). The cells were then washed with PBS and fixed in 2.5 % glutaraldehyde and 2 % paraformaldehyde at room temperature. Subsequently, the samples were stained with uranyl acetate and lead citrate and photographed by Hitachi H7650 microscope.

### 2.17 Immunoblotting

GBM cells were treatment with different concentrations of BS1801, MKC8866 (1 μM), GSK2606414 (1 μM) and NAC (5 mM) for 24 h. Cell lysis buffer (Sigma-Aldrich) with 1 % PMSF and 1 % phosphatase inhibitor cocktail (Selleck) were added to cells to extract proteins. After centrifuging at 13,000 r/min for 20 min, supernatants were collected and heated with 5 × loading buffer. Proteins were separated by SDS-PAGE and electrically transferred to PVDF membrane. The membrane was subsequently blocked with 5 % non-fat milk at room temperature for 1 h. After washing, the membrane was incubated with primary antibodies (Supplementary Table S3) at 4 °C overnight. The membrane was sequentially incubated with HRP-conjugated secondary antibodies, visualized with ECL substrate reagent (Bio-Rad) and imaged by Universal Hood II.

### 2.18 JC-1 staining

Mitochondrial membrane potential (MMP) was assessed using JC-1 MitoMP Detection kit (Dojindo). 5 × 10^5^ GBM cells were seeded into 6-well plates and treated for 24 h. After washing with PBS, 500 μL 2 μmol/L JC-1 working solution was added into each well and incubated for 30 min. The supernatant was then removed, and the cells were washed three times with HBSS. Finally, 500 μL Imaging Buffer Solution was added and the cells were observed. An increase in the ratio of green/red fluorescence intensity was observed upon mitochondrial depolarization.

### 2.19 Seahorse assay

Oxygen consumption rate (OCR) and extracellular acidification rate (ECAR) were determined using Agilent Seahorse XFe24 Analyzer and Cell Mito Stress Test Kit (Agilent Technology). GBM cells (3 × 10^4^ per well) were seeded into XF24 culture plates and treated with BS1801 or NAC for 24 h at 37 °C with 5 % CO_2_. Then the cells were washed and cultured with un-buffered medium for 2 h at 37 °C in CO_2_-free incubator. After adding oligomycin, proximity compound carbonyl cyanide 4-(trifluoromethoxy) phenylhydrazone (FCCP), and rotenone/antimycin A into loading slot, OCR and ECAR were calculated automatically via Seahorse Wave software (version 2.6.3). Basal respiration, ATP production and max respiration were shown in exporting result.

### 2.20 Subcutaneous GBM xenograft model

Female BALB/c nude mice (6 weeks) were purchased from the VitalRiver Animal Lab (Beijing, China). 2 × 10^6^ GBM cells were subcutaneously injected into mice armpits. When tumors grew to an average volume of 20 mm^3^, mice were randomly grouped and orally treated with different drugs every day. Tumor volumes and weights were measured every three days with hand-held vernier calipers and weighting scale. Tumor volumes were calculated with the following formula: volume = (length × width × width)/2. After treatment for several days, mice were euthanatized and tumors were harvested to calculate inhibition rate of various drugs. Meanwhile, main internal organs of mice were also collected, and the organ coefficients were calculated as indicators of drug-induced organ toxicity. U87 tumor tissues were embedded in paraffin and cut into 5 μm sections. Samples were deparaffinized in an oven at 65 °C for 3 h. Then the samples were rehydrated in decreasing concentrations of alcohol. After antigen retrieving and blocking, anti-CHOP antibody was added to the sections and samples were incubated overnight at 4 °C. Following standard immunohistochemistry (IHC) procedures, representative photos were taken with an optical microscope.

### 2.21 Orthotopic GBM xenograft models

Female NOD-Prkdc^scid^ IL2rg^null^ immunodeficient mice were purchased from VitalStar Animal Lab (Beijing, China). After adequate anesthesia, 2 × 10^5^ BNI-19-1-luciferase cells were stereotactically injected into the left frontal lobe of mice. The mice were randomly assigned to receive daily intraperitoneal injections of different drugs. After 5 weeks treatment, drugs were withdrawal and observation period started. Living imaging was conducted per week to measure the tumor volume until week six. Survival observations continued until week 8. Animal survival rates were calculated using GraphPad Software (version 9.0). The endpoint of experiment was a moribund state or death of the animal in accordance with the Institutional Animal Welfare Regulations.

### 2.22 GBM organoids establishment and frozen section staining

The GBM organoids were derived from GBM patients in Beijing Tiantan Hospital and established in our lab. Isolated GBM tumors were cut into small pieces and suspended in medium with N2- and B27-containing media (N2 medium: DMEM/F12 GlutaMAX, 1 × N2, 5 μg/ml insulin, 1 mM l-glutamine, 1 × non-essential amino acids, 100 μM 2-mercaptoethanol; B27 medium: Neurobasal, 1 × B27, 200 mM l-glutamine) supplemented with 50 U/mL penicillin and streptomycin. The GBM organoids were treated with different concentrations of BS1801 for 24 h and then fixed with 4 % paraformaldehyde. After gradient dehydration with sucrose solution for 24 h, organoids were embedded by OCT and then frozen in liquid nitrogen. Tissues were sectioned into 20 μm slices. Anti-CHOP and Cleaved Caspase-3 were added to sections and incubated overnight at 4 °C. After washing with PBST, the slices were incubated with secondary antibodies at room temperature for 1 h. Prolong™ Diamond Antifade Mountant with DAPI (Invitrogen) was added to the dishes, and photos were taken with confocal microscopy.

### 2.23 Blood-Brain-Barrier permeability measurement

MDCK-MDR1 cells were diluted to 1.5 × 10^6^ cells/mL with culture medium and 50 μL cell suspension was dispensed into the filter well of the 96-well HTS Transwell plate. Cell culture medium was replaced every other day. 10 mM stock solutions of BS1801, BS1801-nano and control compounds were prepared. Metoprolol and imatinib were used as control compounds. 50 μL of samples were transferred from donor sides to a new 96-well plate containing 250 μL quenching solvents, vortexed at 1000 rpm for 5min, and then centrifuged at 4000 rpm for 20 min. An aliquot of 100 μL supernatant was mixed with 100 μL pure waters for LC/MS/MS analysis. The apparent permeability coefficient (Papp) for MDCK-MDR1 drug transport assays was calculated using the following equation: Papp = (VA × [drug]acceptor)/(Area × Time × [drug]initial, donor).

### 2.24 Statistical analysis

R software (version 4.2.0) and GraphPad Prism (version 9.0) were used for graphic generation and statistical analyses. Data collected from at least three independent experiments were expressed as mean ± standard deviation (SD). P < 0.05 was considered to be statistically significant.

## 3 Results

### 3.1 Association of oxidative stress status with TMZ resistance in glioma

CGGA database (n = 325) and CGGA (2019) database (n = 693) were used as discovery database and validation database to elucidate the mechanism in glioma recurrence. GSVA was conducted to calculate the score of each biological function for every glioma patient. All biological functions were divided into four groups according to primary or recurrent tumor status. We established further screening criteria that the biological function had a higher score in recurrent patients and a p-value of less than 0.05 in both databases. Combined with the significant difference of p-values in the database containing the most recurrent glioma patients, we determined “Response to Oxidative Stress” was the most significant function (Fig. 1A and Table 1). Thus, we hypothesized that the status of oxidative stress in glioma cells may contribute to TMZ resistance.

To further validate our assumption, we used TMZ to treat LN229, U87 and U251 cells to establish TMZ resistant cell lines in vitro (Fig. S1A–D). Among this process, IF and Western blot assays were performed to detect the expression levels of γ-H2AX, which is a sensitive indicator to reflect the degree of DNA double-strand damage. We found that the maximum degree of damage was occurred after TMZ 72 h treatment in LN229, U87 and U251 cell lines (Fig. 1B–E and S1E–F). Meanwhile, the expression levels of γ-H2AX were decreased with the extension of TMZ treatment, indicating that glioma cells develop TMZ resistance. (Fig. S1G–H). Besides, as the production of oxidative stress, ROS levels were detected by flow cytometry in different stages of TMZ treatment. As expected, consistent with the trend of γ-H2AX expression levels, the ROS levels in glioma cells also decreased significantly after TMZ treatment (Fig. 1F–G and S1I).

To investigate the underlying mechanism of TMZ resistance, RNA sequencing was performed in TMZ-WT, TMZ-I and TMZ-R LN229 cells (Fig. 1H). Among the oxidative stress correlated genes, we found that the expression levels of *ATF4*, *HMOX1*, *PLK3*, *PTGS2*, *SOD2*, *SRXN1*, *STC2*, *TNFAIP3* and *TXNRD1* were significantly increased after TMZ treatment (Fig. 1I). Subsequently, we further analyzed and validated the genes with significantly increased expression in recurrent glioma patients compared to primary glioma patients in both databases, and intersected them with the above 9 genes. Finally, we found that only *TXNRD1* showed increasing expression level with statistically significant differences in three databases. (Fig. 1J). Since *TXNRD1* is a gene encoding thioredoxin reductase 1 (TrxR1), which can maintain the reduction level of cells [32], we speculate that *TXNRD1* is closely related to TMZ resistance in glioma.

### 3.2 Correlation between *TXNRD1* and malignant biological events in glioma

**Supplementary Fig. S2:**
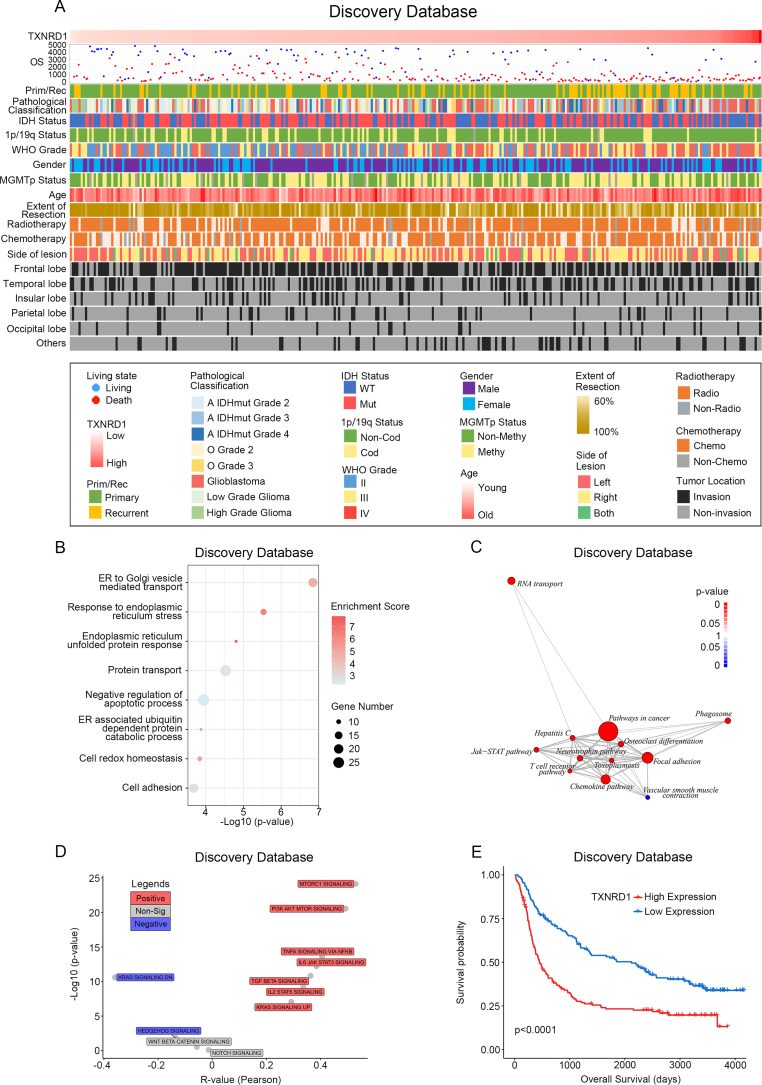
*TXNRD1* was associated with malignancy biological events in glioma in discovery database. (A) Heatmap showed *TXNRD1* was closely associated with malignancy molecular markers. (B) GO analysis showed that *TXNRD1* related genes were mostly associated with ER stress and redox homeostasis. (C) KEGG pathway analysis showed that *TXNRD1* related genes were mostly involved in cancer pathways and RNA transport. (D) Pearson correlation analysis showed that *TXNRD1* was positively associated with proliferation and inflammation pathways. (E) Kaplan-Meier survival analysis showed that patients with high expression of *TXNRD1* had a reduced survival time.

**Supplementary Fig. S3:**
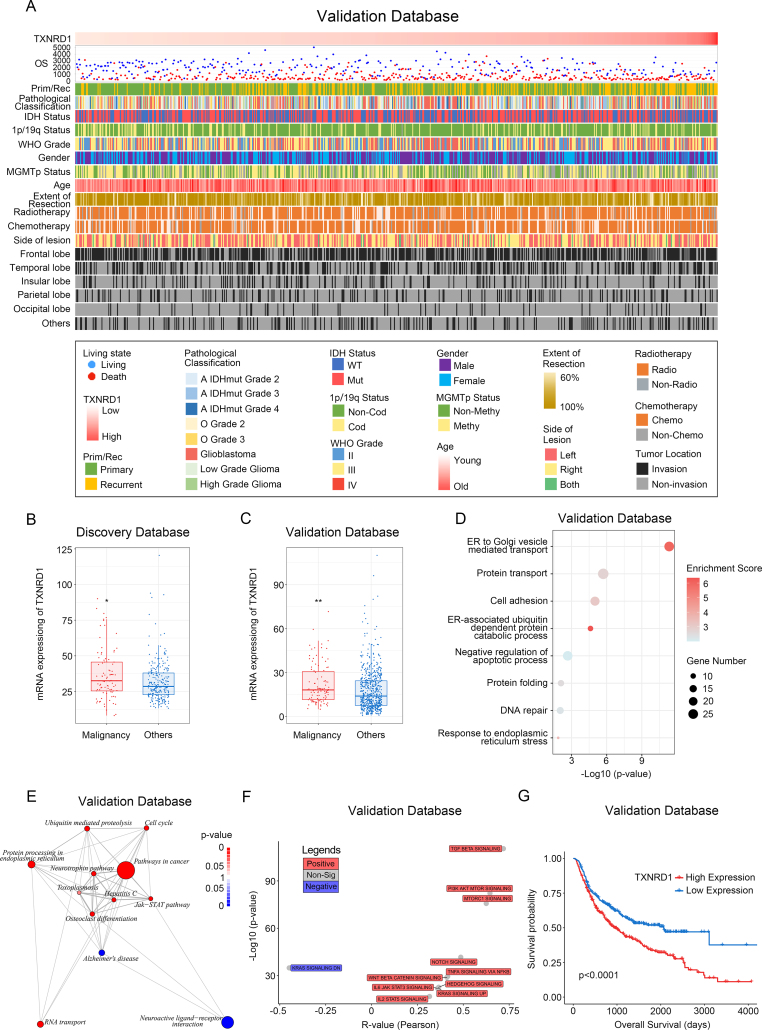
*TXNRD1* was associated with malignancy biological events in glioma in validation database. (A) Heatmap showed *TXNRD1* was closely associated with malignancy molecular markers. (B–C) Boxplot showed that *TXNRD1* expression was much higher in Malignancy group than others in both databases. Malignancy: glioma patients with IDH wildtype, 1p/19q non-codeletion and MGMT promoter unmethylation. (D) GO analysis showed that *TXNRD1* related genes were mostly associated with ER stress and redox homeostasis. (E) KEGG pathway analysis showed that *TXNRD1* related genes were mostly involved in cancer pathways and RNA transport. (F) Pearson correlation analysis showed that *TXNRD1* was positively associated with proliferation and inflammation pathways. (G) Kaplan-Meier survival analysis showed that patients with high expression of *TXNRD1* had a reduced survival time. ∗ and ∗∗ indicated that p < 0.05 and p < 0.01, respectively.

To clearly visualize the association between *TXNRD1* and known malignant biomarkers in glioma patients, landscapes were drawn in discovery and validation databases, respectively (Fig. S2A and S3A). The expression of *TXNRD1* was significantly higher in IDH wildtype, 1p/19q non-codeletion, MGMT unmethylation glioblastoma patients in both databases (Fig. S3B–C). To further investigate *TXNRD1*-related biological functions in gliomas, we performed GO and KEGG analysis. As expected, genes that associated with *TXNRD1* were involved in oxidative stress mediated functions, including endoplasmic reticulum stress, cell redox homeostasis, endoplasmic reticulum to Golgi protein transport and ubiquitin-dependent protein catabolic process in both databases (Fig. S2B and S3D). Additionally, KEGG pathway analysis identified these genes to be positively associated with growth and inflammatory pathways, including MTORC1, PI3K/AKT/MTOR, TNF-α, TGF-β, IL6/JAK/STAT3 signaling (Fig. S2C–D and S3E-F). Meanwhile, patients with higher *TXNRD1* expression level also had worse prognosis (Fig. S2E and S3G).

### 3.3 Inhibition of BS1801 on TrxR1 activity and glioma cell growth

**Supplementary Fig. S4:**
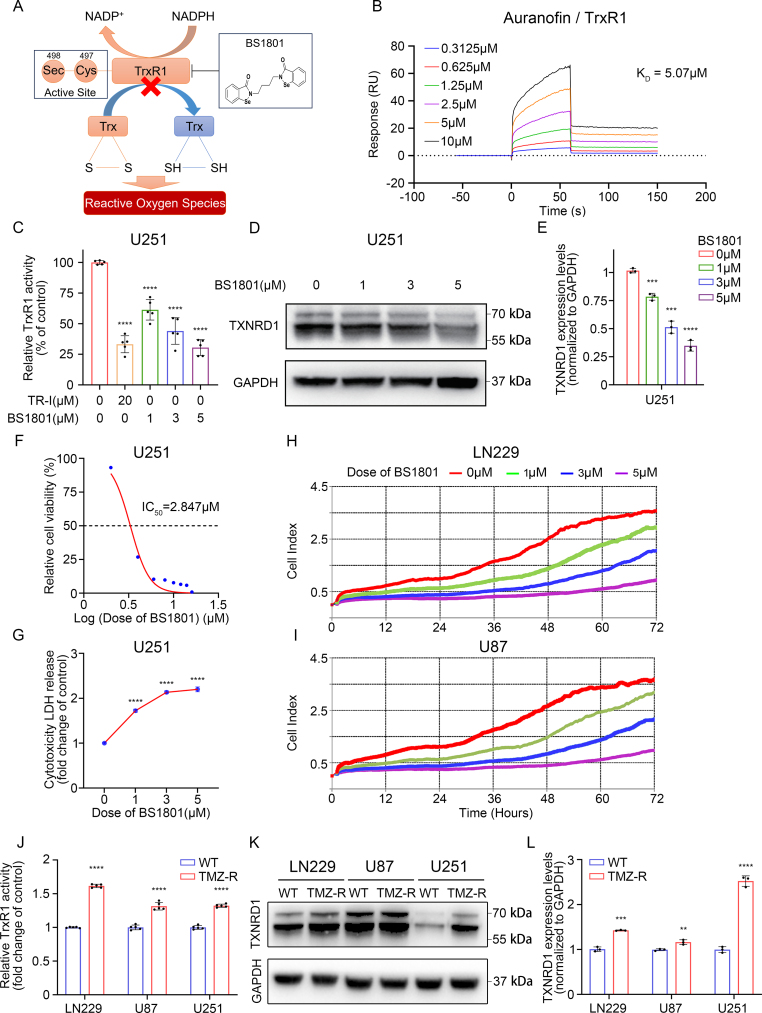
BS1801 inhibited TrxR1 activity and glioma cell growth. (A) The workflow of BS1801 inhibition on thioredoxin system. BS1801 interacted with TrxR1 at Cys497/Sec498 active site and competitively inhibited TrxR1 enzyme activity. Oxidative TrxR1 lost its reducing ability, resulting in increased oxidative stress and elevated ROS level. (B) The binding affinity of auranofin with recombination human TrxR1 protein were determined by using SPR assay. K_D_: dissociation constant. (C) DTNB assay revealed that the TrxR1 inhibition rates after BS1801 treatment on U251 cell line for 24 h. TR-I was a strong TrxR inhibitor and set as positive control. N = 5 for each group. (D-E) Immunoblots for TXNRD1 in U251 cell line after BS1801 treatment for 24 h. GAPDH was probed as loading control. (F) CCK8 test detected the cell viability after different concentrations of BS1801 treatment inU251 cell line for 24 h. (G) Cytotoxicity LDH release assay measured LDH contents after different concentrations of BS1801 treatment in U251 cell line for 24 h. (H–I) RTCA test detected cell index after BS1801 treatment in LN229 and U87 cell lines. (J) DTNB assay revealed that the relative TrxR1 activity in wildtype and TMZ resistant glioma cell lines. N = 5 for each group. (K-L) Immunoblots for TXNRD1 in wildtype and TMZ resistant glioma cell lines. GAPDH was probed as loading control. N = 3 for each group. ∗∗, ∗∗∗ and ∗∗∗∗ indicated that p < 0.01, p < 0.001 and p < 0.0001, respectively.

Based on above results, we hypothesized that inhibiting TrxR1 may disturb glioma cells survival through elevating oxidative stress levels. Therefore, a novel small-molecular compound BS1801 containing organoselenium was designed to interact with TrxR1 at Cys497/Sec498 active site and competitively inhibit TrxR1 activity, resulting in stronger oxidative stress (Fig. S4A). Surface Plasmon Resonance (SPR) assays were performed to explore the interaction between BS1801 and TrxR1. The result showed that the affinity constant K_D_ value of BS1801/TrxR1 was 2.44 μM (Fig. 2A). In comparison, the K_D_ value of another well-known TrxR1 inhibitor auranofin was 5.07 μM (Fig. S4B), indicated that BS1801 has a stronger binding affinity to TrxR1. Subsequently, DTNB assay revealed that TrxR1 activity was significantly decreased in a dose-dependent manner in LN229 (Fig. 2B), U87 (Fig. 2C) and U251 (Fig. S4C) cells after BS1801 treatment for 24 h. Consistently, the protein expression level of TXNRD1 was also decreased after BS1801 treatment (Fig. 2D–E and S4D-E). All these findings suggested that BS1801 inhibited TrxR1 activity and expression level in a dose-dependent manner.

Additionally, we investigated whether BS1801 disturbed glioma cells survival. We found that BS1801 inhibited the proliferation of glioma cell lines LN229 (Fig. 2F), U87 (Fig. 2G), BNI-19-1 (Fig. 2H), BNI-20-1 (Fig. 2I) and U251 (Fig. S4F) in a dose-dependent manner. The IC_50_ values of the five cell lines were around 2 μM. LDH, which released in medium after cell fracturing, was dramatically increased after 5 μM BS1801 treatment (Fig. 2J–M and S4G). Moreover, the real-time cell growth curve also exhibited strong proliferative inhibition effect of BS1801 (Fig. S4H–I).

**Supplementary Fig. S5:**
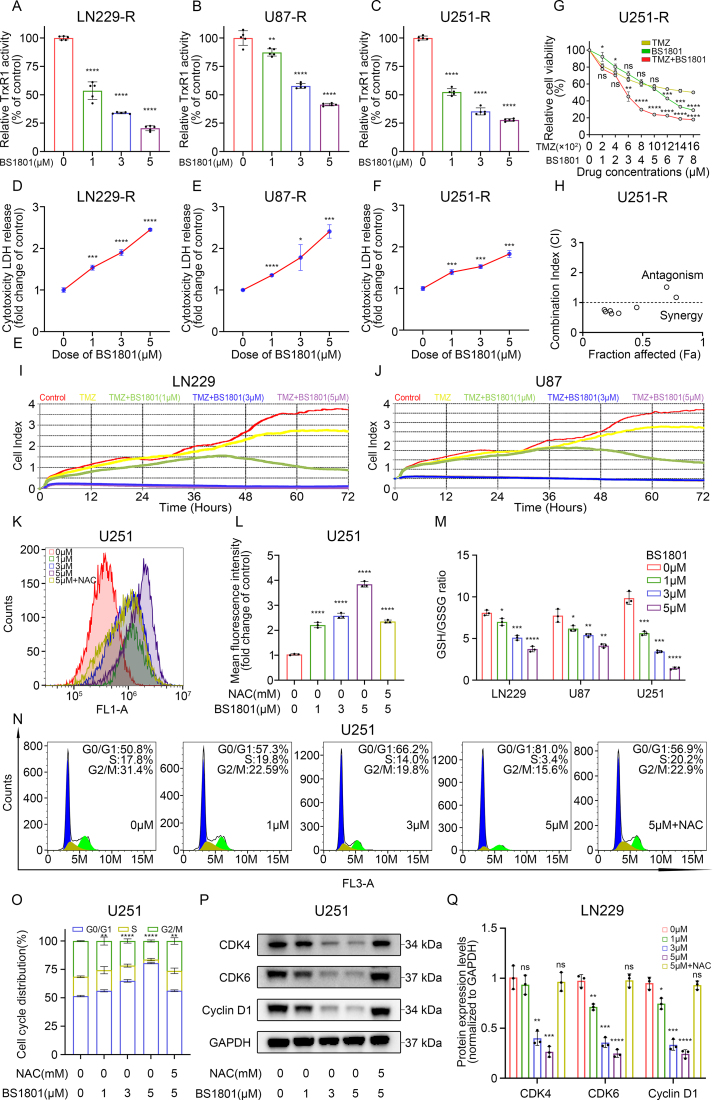
BS1801 synergized with TMZ in vitro and induced cell cycle arrest. (A-C) DTNB assay revealed that the TrxR1 inhibition rates after BS1801 treatment in TMZ resistant cell lines for 24 h. (D-F) Cytotoxicity LDH release assay measured LDH contents after different concentrations of BS1801 treatment in TMZ resistant cell lines for 24 h. (G-H) CCK8 test detected the cell viability after different concentrations of BS1801 and TMZ treatment in TMZ resistant U251 cell lines. TMZ: BS1801 = 200: 1. Fa-CI curve reflected synergy or antagonism effect of BS1801 and TMZ at different concentrations in TMZ resistant cell lines. (I-J) RTCA test detected cell index after different concentrations of BS1801 treatment under 200 μM TMZ condition in LN229 and U87 cell lines. (K-L) Flow cytometry detected ROS levels after BS1801 with or without NAC treatment in U251 cell line for 24 h. (M) GSH/GSSG-GloTM Assay detected GSH/GSSG ratio after BS1801 treatment in LN229, U87 and U251 cell lines for 24 h. (N–O) Representative images and histogram analysis of G0/G1 arrest in U251 cell line after BS1801 with or without NAC treatment for 24 h. (P-Q) Immunoblots for CDK4, CDK6 and Cyclin D1 after BS1801 with or without NAC treatment in U251 cell line for 24 h. GAPDH was probed as loading control. N = 3 for each group. ns, ∗, ∗∗, ∗∗∗ and ∗∗∗∗ indicated that no significance, p < 0.05, p < 0.01, p < 0.001 and p < 0.0001, respectively.

To determine the effect of BS1801 on TMZ resistant glioma cells, we further performed in vitro experiments in LN229-R, U87-R and U251-R cells. Results suggested that the protein expression level and activity of TrxR1 were relatively higher in TMZ resistant glioma cells compared with wildtype (Fig. S4J–L). Subsequently, we found that BS1801 also inhibited TrxR1 activity and elevated LDH levels in a dose-dependent manner in TMZ resistant glioma cells (Fig. S5A–F), suggesting that BS1801 may eliminate these glioma cells to relieve TMZ resistance. Therefore, Fa-CI analysis was further performed to investigate the synergy effect of TMZ and BS1801. Results showed that BS1801 synergized with TMZ when BS1801 > 2 μM and TMZ >400 μM in LN229-R, U87-R and U251-R cells (Fig. 2N–Q and S5G-H). Real-time detection also showed that 3 μM or 5 μM BS1801 treatment could inhibit LN229 and U87 cells proliferation all the time under 200 μM TMZ condition (Fig. S5I–J).

### 3.4 BS1801 elevated ROS level and induced glioma cells apoptosis

**Fig. 3:**
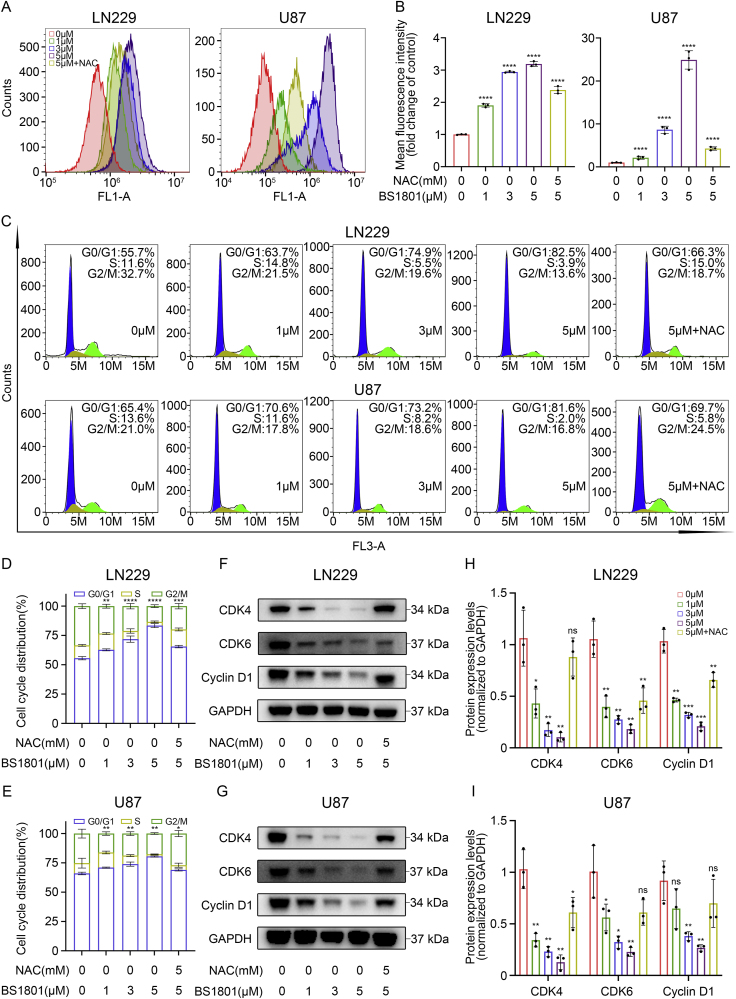
**BS1801 elevated ROS level and induced cell cycle arrest.** (A–B) Flow cytometry detected ROS levels after BS1801 with or without NAC treatment in LN229 and U87 cell lines for 24 h. (C–E) Representative figures and histogram analysis of G0/G1 arrest in LN229 and U87 cell lines after BS1801 with or without NAC treatment for 24 h. (F–I) Immunoblots for CDK4, CDK6 and Cyclin D1 after BS1801 with or without NAC treatment in LN229 and U87 cell lines for 24 h. GAPDH was probed as loading control. N = 3 for each group. ns, ∗, ∗∗, ∗∗∗ and ∗∗∗∗ indicated that no significance, p < 0.05, p < 0.01, p < 0.001 and p < 0.0001, respectively.

To further explore the anti-glioma mechanisms of BS1801, intracellular ROS levels were firstly evaluated. We found that DCF mean fluorescence intensity was dose-dependently increased after BS1801 treatment in glioma cells. Meanwhile, the reductive agent NAC could scavenge ROS and rescue the ROS levels caused by BS1801 (Fig. 3A–B and S5K–L). GSH is another redox mediated system, and low ratio of intracellular GSH to GSSG reflects high ROS content [33]. We then measured the content of GSH and GSSG, and found that the ratio was significantly decreased with the increase of BS1801 concentration in LN229, U87 and U251 cells (Fig. S5M).

**Fig. 4:**
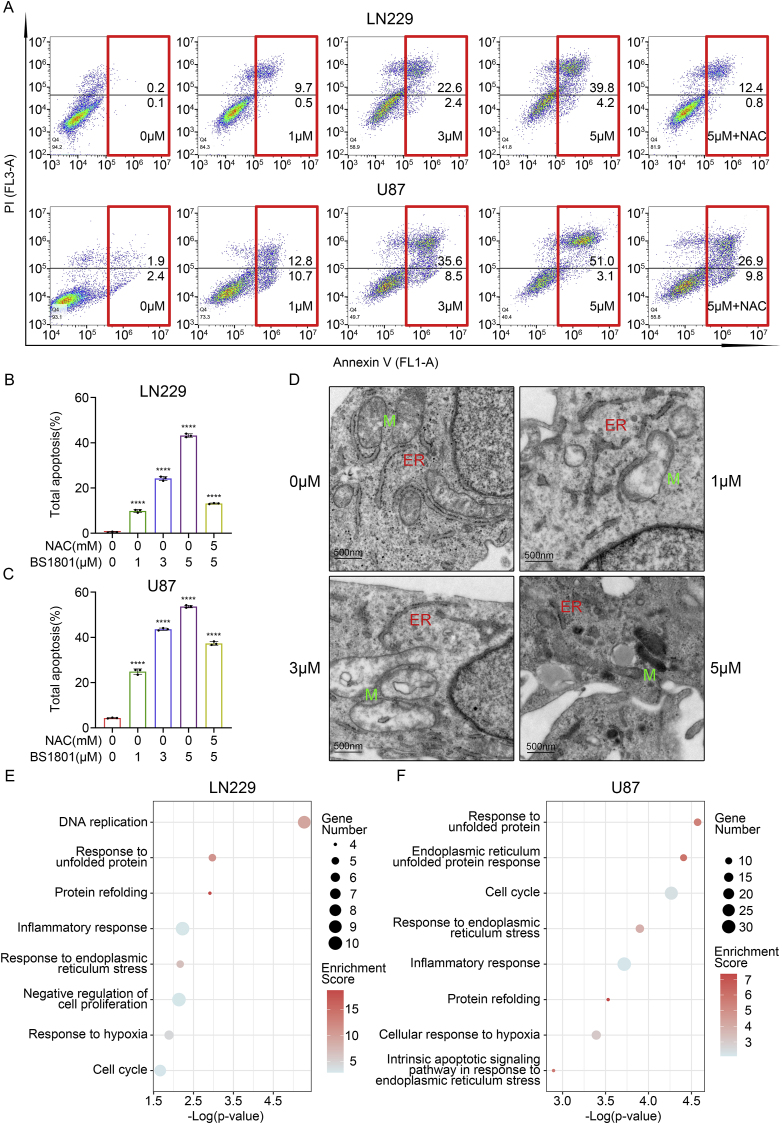
**BS1801 induced cell apoptosis and damaged normal structure of ER and mitochondria.** (A–C) Annexin V-PI analysis and statistical analysis of total apoptosis rate after BS1801 with or without NAC treatment in LN229 and U87 cell lines for 24 h. (D) TEM showed the organelle structure of LN229 cells after BS1801 treatment for 24 h. M: mitochondria; ER: endoplasmic reticulum. Scale bar was 500 nm. (E–F) GO analysis showed that upregulated genes after 5 μM BS1801 treatment were mostly associated with ER stress and response to UPR in LN229 and U87 cell lines. N = 3 for each group. ∗∗∗∗ indicated that p < 0.0001.

**Supplementary Fig. S6:**
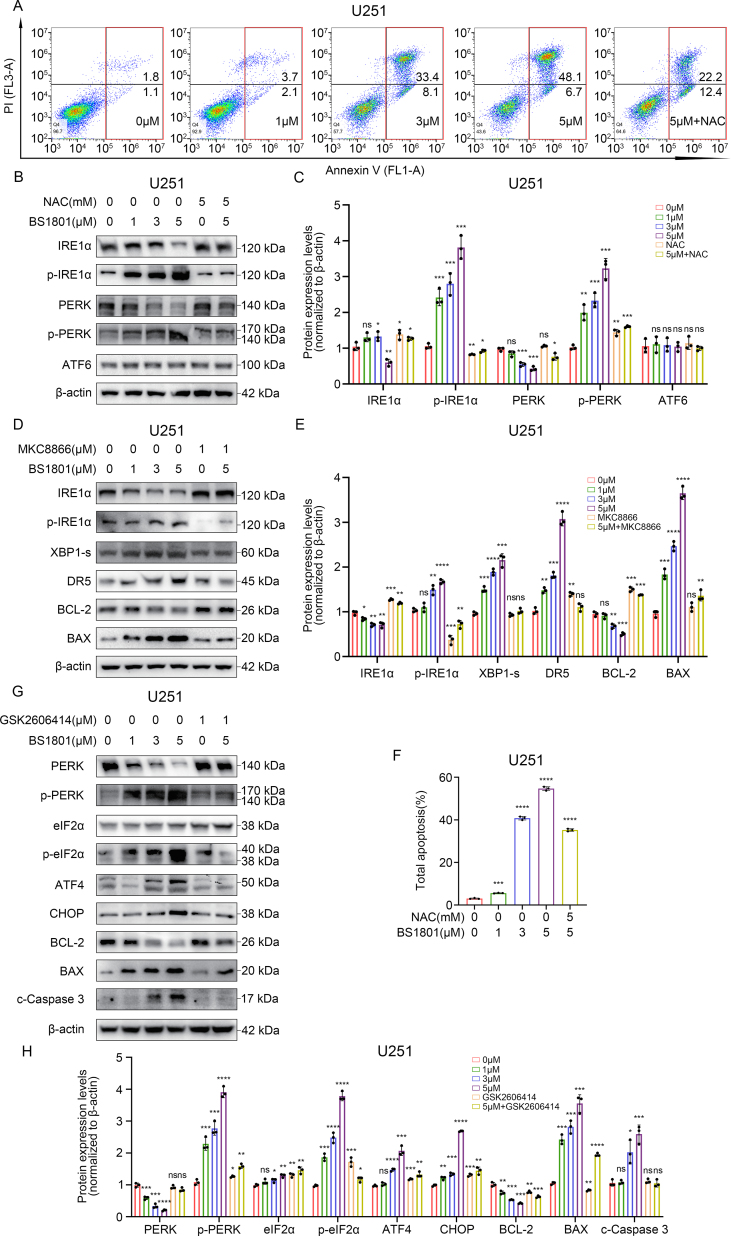
BS1801 induced ER stress and apoptosis in glioma cells. (A, F) Annexin V-PI analysis and statistical analysis of total apoptosis rate after BS1801 with or without NAC treatment in U251 cell line for 24 h. (B–C) Immunoblots for IRE1α, p-IRE1α, PERK, p-PERK and ATF6 after BS1801 with or without NAC treatment for 24 h in U251 cell line. β-actin was probed as loading control. (D-E) Immunoblots for IRE1α, p-IRE1α, XBP1s, DR5, BCL-2 and BAX after BS1801 with or without MKC8866 treatment for 24 h in U251 cell line. β-actin was probed as loading control. (G-H) Immunoblots for PERK, p-PERK, eIF2α, *p*-eIF2α, ATF4, CHOP BCL-2, BAX and Cleaved Caspase-3 after BS1801 with or without GSK2606414 treatment for 24 h in U251 cell line. β-actin was probed as loading control. N = 3 for each group. ns, ∗, ∗∗, ∗∗∗ and ∗∗∗∗ indicated that no significance, p < 0.05, p < 0.01, p < 0.001 and p < 0.0001, respectively.

Subsequently, considering the anti-proliferation ability of BS1801, we performed flow cytometry analysis to detect the changes of cell cycle after treatment. It was obvious that LN229, U87 and U251 cells in G0/G1 phase were significantly augmented with the increase in BS1801 concentration (Fig. 3C–E and S5N–O). Likewise, the cell cycle proteins essential for promoting cell cycle from G1 to S phase, including CDK4, CDK6 and Cyclin D1, were all decreased after BS1801 treatment in a dose-dependent manner (Fig. 3F–I and S5P-Q). Besides, BS1801 treatment dose-dependently induced total apoptosis in LN229, U87 and U251 (Fig. 4A–C and S6A, F) cells. The G0/G1 cell cycle arrest and cell apoptosis could be partially rescued by NAC.

### 3.5 BS1801 induced ER stress in glioma cells

Based on the results of GO and flow cytometry, we speculated that BS1801 may induce ER stress through ROS in glioma cells. To validate our hypothesis, we used TEM to observe the morphology of organelles. Results showed that untreated cells had intact subcellular structure with abundant ER and normal morphology. After BS1801 treatment for 24 h, the ER significantly expanded and formed numerous cytoplasmic vacuoles. Additionally, we also observed swollen mitochondria, with fractured or absent mitochondrial cristae (Fig. 4D). These findings suggested that BS1801 damaged normal structure of ER and mitochondria, and may influenced their functions.

**Fig. 5:**
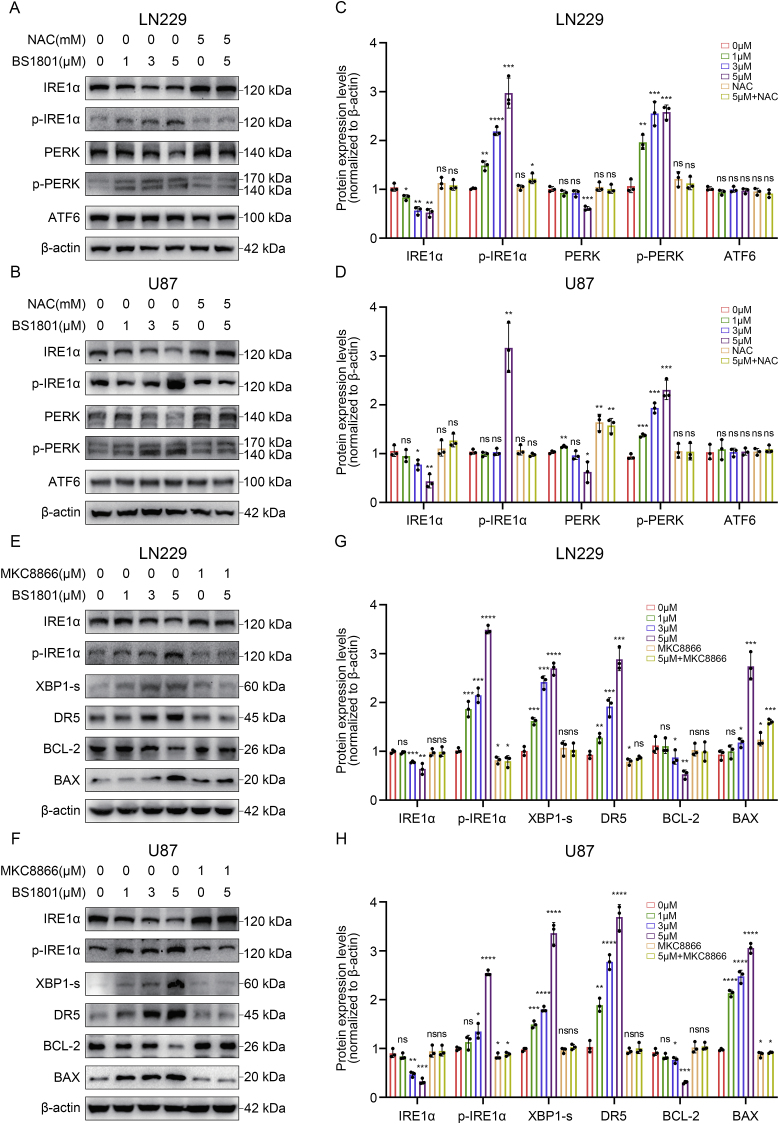
**BS1801 induced ER stress in glioma cells.** (A–D) Immunoblots for IRE1α, p-IRE1α, PERK, p-PERK and ATF6 after BS1801 with or without NAC treatment for 24 h in LN229 and U87 cell lines. β-actin was probed as loading control. (E–H) Immunoblots for IRE1α, p-IRE1α, XBP1s, DR5, BCL-2 and BAX after BS1801 with or without MKC8866 treatment for 24 h in LN229 and U87 cell lines. β-actin was probed as loading control. N = 3 for each group. ns, ∗, ∗∗, ∗∗∗ and ∗∗∗∗ indicated that no significance, p < 0.05, p < 0.01, p < 0.001 and p < 0.0001, respectively.

RNA-seq was conducted to investigate the biological functions involved in glioma cells after BS1801 treatment. The GO results revealed that upregulated genes were participated in several ER associated functions, including response to unfolded protein, response to ER stress, ER unfolded protein response and response to hypoxia, suggesting that BS1801 treatment may trigger ER stress in glioma cells (Fig. 4E–F). Evidence has demonstrated that unfolded protein response (UPR) was triggered by ER stress and mainly through three signaling pathways to maintain ER functions [34]. Then we investigated the expression of three conserved signal transducers in UPR. The Western blot results showed that IRE1α and PERK signaling pathways may be activated after BS1801 treatment, while no obvious change was found in ATF6 signaling pathway in LN229, U87 and U251 cells (Fig. 5A–D and S6B–C). As expected, NAC eliminated the changes caused by BS1801, suggested that BS1801 may induce ER stress by ROS.

**Fig. 6:**
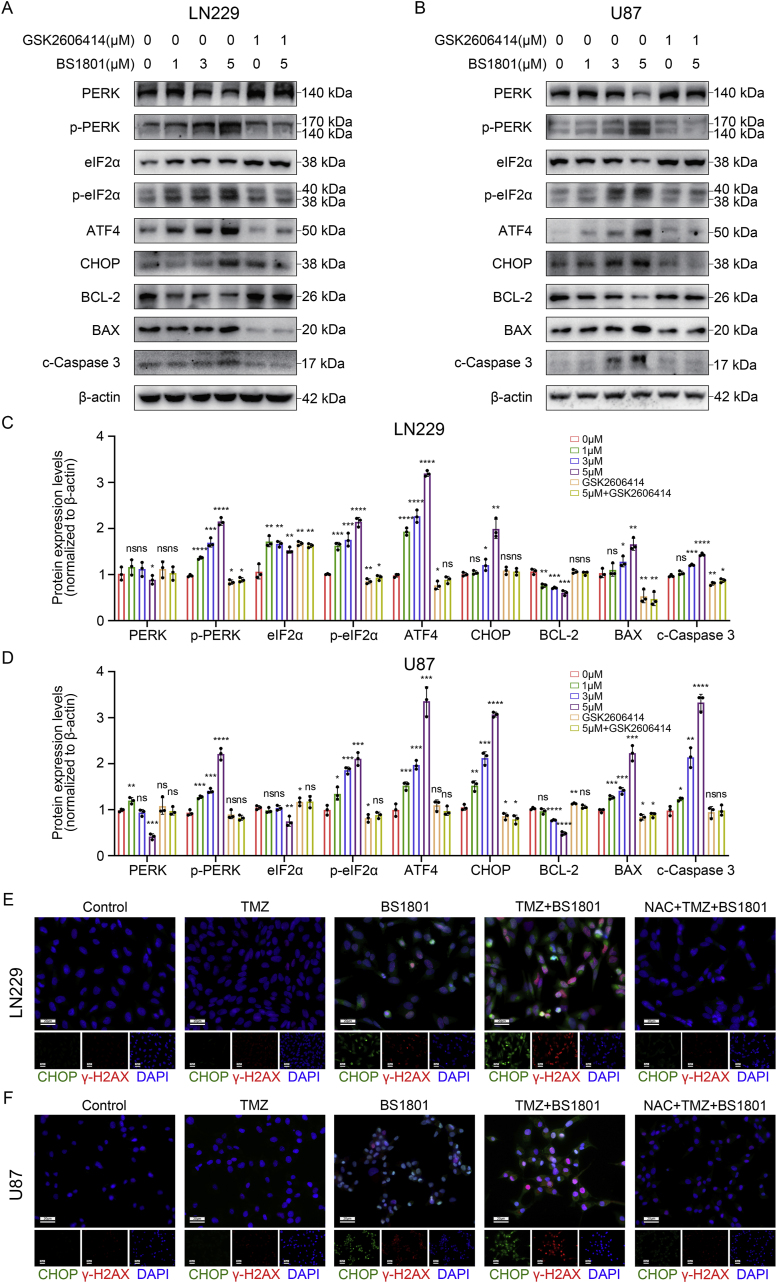
**BS1801 induced cell apoptosis by activating PERK signaling pathway.** (A–D) Immunoblots for PERK, p-PERK, eIF2α, p-eIF2α, ATF4, CHOP BCL-2, BAX and Cleaved Caspase-3 after BS1801 with or without GSK2606414 treatment for 24 h in LN229 and U87 cell lines. β-actin was probed as loading control. (E–F) Representative IF figures showed γ-H2AX and CHOP staining in LN229 and U87 cell lines after different drugs treatment. Red fluorescence represented γ-H2AX. Green fluorescence represented CHOP. Cell nuclei were stained with DAPI. Scale bar was 20 μm. N = 3 for each group. ns, ∗, ∗∗, ∗∗∗ and ∗∗∗∗ indicated that no significance, p < 0.05, p < 0.01, p < 0.001 and p < 0.0001, respectively.

**Supplementary Fig. S7:**
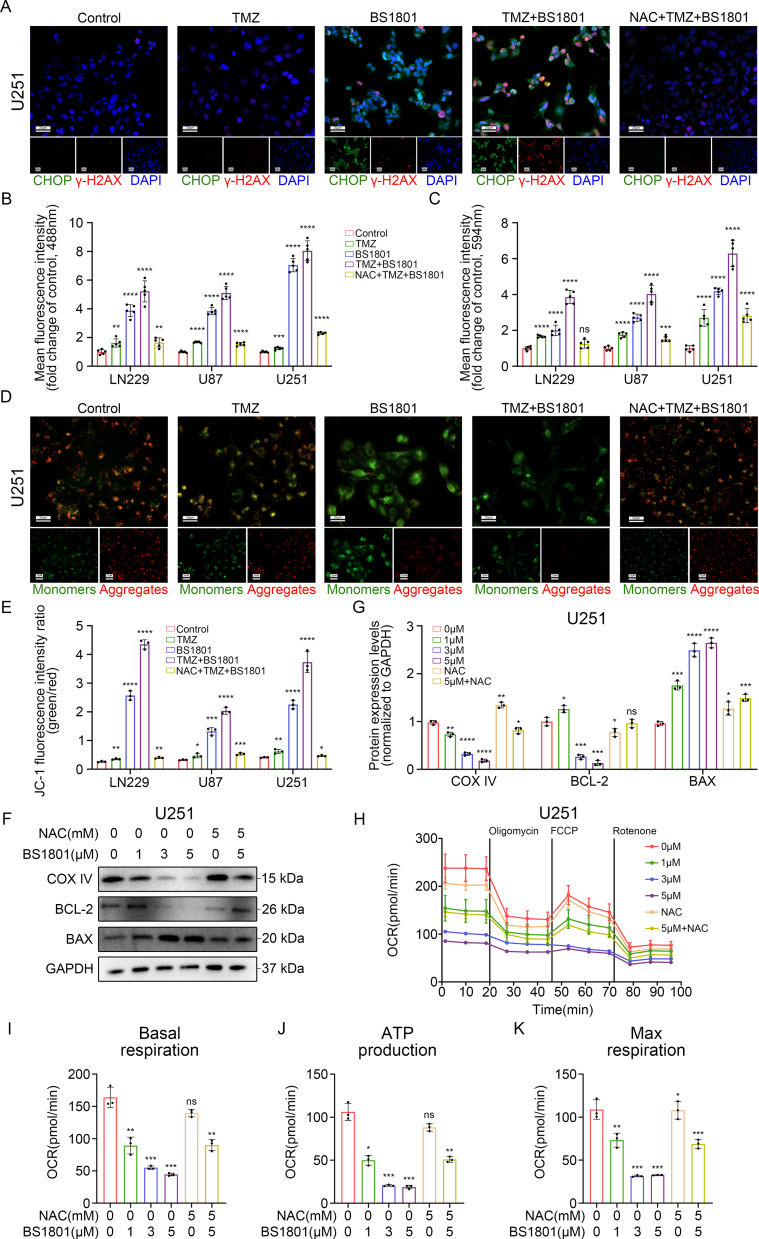
BS1801 induced mitochondrial dysfunction in glioma cells. (A-C) Representative IF images and histogram analysis of γ-H2AX and CHOP staining in U251 cell line after different drugs treatment. Red fluorescence represented γ-H2AX. Green fluorescence represented CHOP. Cell nuclei were stained with DAPI. Scale bar was 20 μm. (D-E) Representative IF images and histogram analysis of JC-1 in U251 cell line after different drug treatments. Red fluorescence represented JC-1 aggregates. Green fluorescence represented JC-1 monomers. Scale bar was 20 μm. (F-G) Immunoblots for COX IV, BCL-2 and BAX after BS1801 with or without NAC treatment for 24 h in U251 cell line. GAPDH was probed as loading control. (H–K) Mitochondrial respiration function was measured through detecting the changes of OCR following oligomycin, FCCP and rotenone treatment. Glioma cells were pre-treated by different concentrations of BS1801 or NAC. N = 3 for each group. ns, ∗, ∗∗, ∗∗∗ and ∗∗∗∗ indicated that no significance, p < 0.05, p < 0.01, p < 0.001 and p < 0.0001, respectively.

Based on these findings, we further explored the downstream changes of IRE1α and PERK signaling pathways after BS1801 treatment. In IRE1α axis, with phosphorylated-IRE1α activating, downstream proteins expression, including XBP1s, DR5 and BAX were upregulated. Meanwhile, anti-apoptosis protein BCL-2 was downregulated. Additionally, IRE1α inhibitor MKC8866 reversed these changes caused by BS1801 (Fig. 5E–H and S6D-E). In PERK axis, phosphorylated-PERK and phosphorylated-eIF2α expression were been upregulated. Then ATF4 and CHOP were been activated. Apoptosis-modulated protein BAX and Cleaved Caspase-3 expression were been upregulated, while the expression of BCL-2 was downregulated. Similarly, PERK inhibitor GSK2606414 reversed these changes caused by BS1801 (Fig. 6A–D and S6G–H). Finally, we examined the expression of γ-H2AX and CHOP after BS1801 treatment and found that the combination of TMZ and BS1801 exhibited the greatest DNA damage degree and ER stress status (Fig. 6E–F and S7A-C). All these results suggested that BS1801 may trigger ER stress in glioma cells by ROS and promote apoptosis through IRE1α and PERK UPR branches.

### 3.6 BS1801 induced mitochondrial dysfunction in glioma cells

**Fig. 7:**
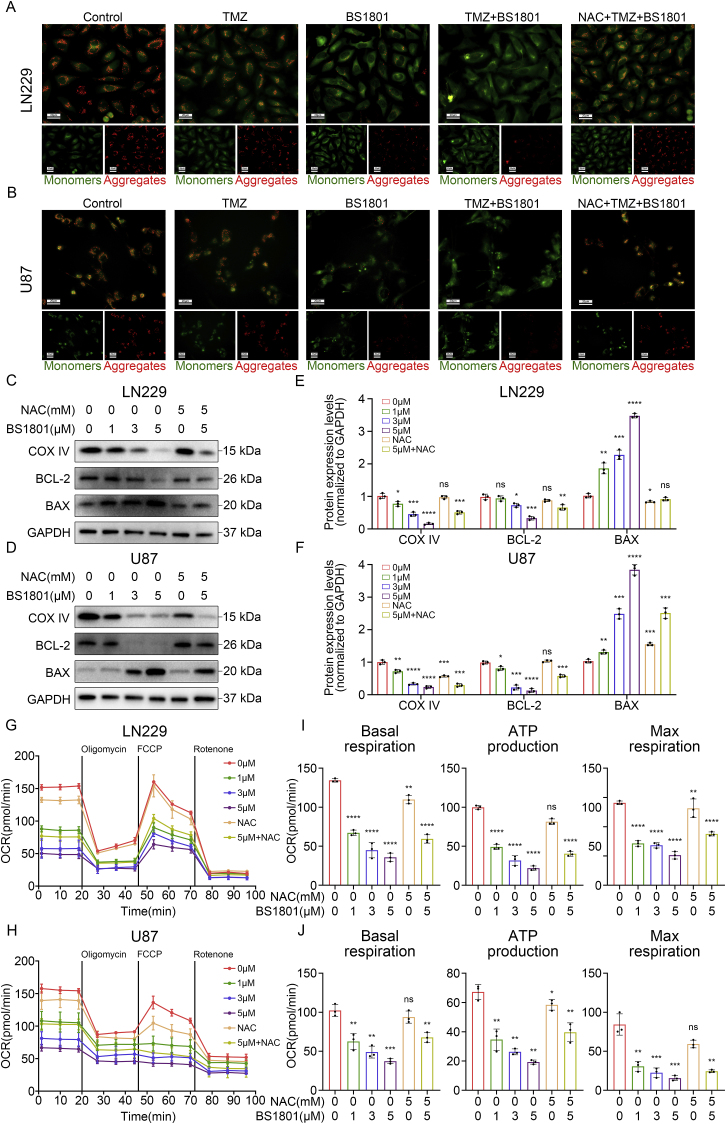
**BS1801 induced mitochondrial dysfunction in glioma cells.** (A–B) Representative IF images of JC-1 probe in LN229 and U87 cell lines after different drug treatments for 24 h. Red fluorescence represented JC-1 aggregates. Green fluorescence represented JC-1 monomers. Scale bar was 20 μm. (C–F) Immunoblots for COX IV, BCL-2 and BAX after BS1801 with or without NAC treatment for 24 h in LN229 and U87 cell lines. GAPDH was probed as loading control. (G–J) Mitochondrial respiration function was measured through detecting the changes of OCR following oligomycin, FCCP and rotenone treatment. Glioma cells were pre-treated by different concentrations of BS1801 or NAC. N = 3 for each group. ns, ∗, ∗∗, ∗∗∗ and ∗∗∗∗ indicated that no significance, p < 0.05, p < 0.01, p < 0.001 and p < 0.0001, respectively.

Since the TEM results showed the effect of BS1801 on mitochondria, we further measured mitochondrial functions after drug treatment in glioma cells. Initially, JC-1 probe was used to assessed the changes of MMP after different treatments. As expected, TMZ combined with BS1801 could strongly induce mitochondrial depolarization and decrease MMP in LN229, U87 and U251 cells, while NAC treatment relieved certain depolarization, suggesting that these changes might be induced by ROS (Fig. 7A–B and S7D–E). Given the reduced MMP and apoptosis caused by BS1801, we then isolated mitochondrial proteins to assess the expression levels of COX IV, BCL-2 and BAX. Unsurprisingly, BS1801 decreased the expression of COX IV and BCL-2, while elevated BAX expression in a dose-dependent manner. Similarly, NAC treatment partially reversed the effects caused by BS1801 (Fig. 7C–F and S7F–G).

Given the crucial of COX IV in mitochondrial respiratory chain and ATP production, we further performed Seahorse assay to assess the mitochondrial OXPHOS function after BS1801 treatment. Results from mitochondrial stress test showed that basal OCR was significantly decreased after BS1801 treatment. Additionally, BS1801 decreased ATP production and maximal respiration in a dose-dependent manner. As expected, NAC treatment partially reversed these changes (Fig. 7G–J and S7H–K).

### 3.7 BS1801 inhibited glioma proliferation combined with TMZ in vivo

**Fig. 8:**
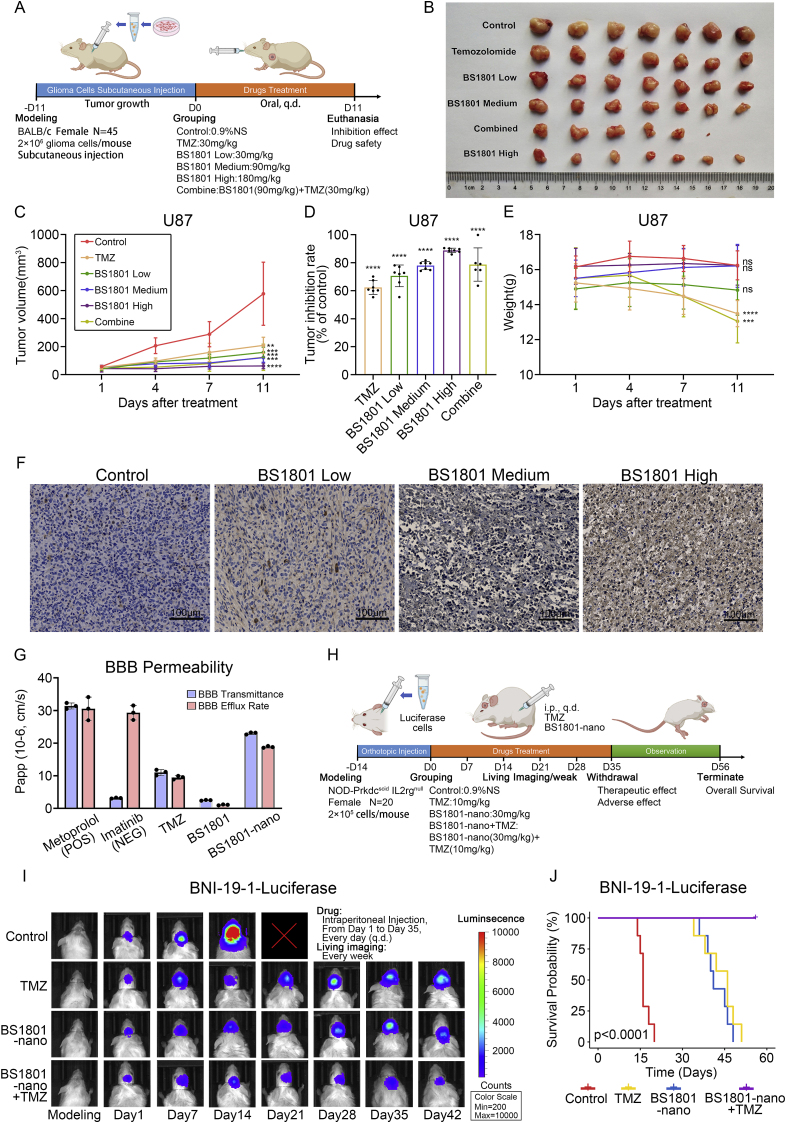
**BS1801 or BS1801-nano inhibited glioma proliferation combined with TMZ in vivo**. (A) The workflow of subcutaneous GBM xenograft models. (B) Tumor morphology of U87 mice models after different drugs treatment. (C) Tumor volume analysis, (D) Tumor inhibition rate analysis and (E) Body weight analysis of different drug treatments. (F) Representative IHC staining of CHOP positive U87 cells after BS1801 treatment. Scale bar was 50 μm. (G) BBB permeability results of control compounds, TMZ, BS1801 and BS1801-nano in MDCK-MDR1 cell line. (H) The workflow of orthotopic GBM xenograft models. (I) Living imaging showed the changes of tumor volume after different drug treatments. (J) Kaplan-Meier survival analysis was performed in different groups. In subcutaneous U87 xenograft models (N = 7 for each group): Control: 0.9 % normal saline; TMZ: 30 mg/kg TMZ treatment; BS1801 Low: 30 mg/kg BS1801 treatment; BS1801 Medium: 90 mg/kg BS1801 treatment; BS1801 High: 180 mg/kg BS1801 treatment; Combine: 30 mg/kg TMZ and 90 mg/kg BS1801 treatment. In orthotopic GBM xenograft models (N = 5 for each group): Control: 0.9 % normal saline; TMZ: 10 mg/kg TMZ treatment; BS1801-nano: 30 mg/kg BS1801-nano treatment; BS1801-nano + TMZ: 30 mg/kg BS1801-nano and 10 mg/kg TMZ treatment. ns, ∗∗, ∗∗∗ and ∗∗∗∗ indicated that no significance, p < 0.01, p < 0.001 and p < 0.0001, respectively.

**Supplementary Fig. S8:**
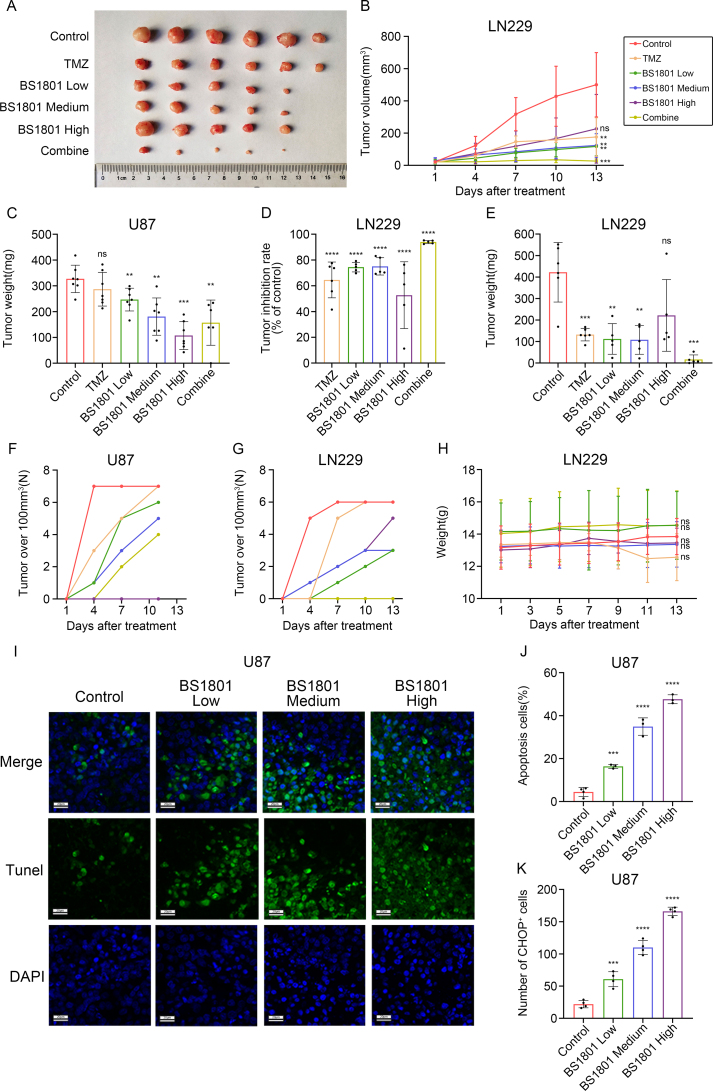
BS1801 inhibited glioma proliferation combined with TMZ in LN229 models. (A) Tumor morphology of LN229 mice models after different drugs treatment. (B) Tumor volume analysis of LN229 models. (C) Tumor weight analysis of U87 models. (D) Tumor inhibition rate analysis of LN229 models. (E) Tumor weight analysis of LN229 models. (F) Tumor growth rate analysis of U87 models. (G) Tumor growth rate analysis of LN229 models. (H) Body weight analysis of different drug treatments in LN229 models. (I-J) Representative IF staining images and histogram analysis of apoptosis U87 cells after BS1801 treatment. Green fluorescence: Tunel. Cell nuclei were stained with DAPI. Scale bar was 20 μm. (K) Histogram analysis of CHOP positive U87 cells after BS1801 treatment. In subcutaneous LN229 xenograft models: Control: 0.9 % normal saline; TMZ: 30 mg/kg TMZ treatment; BS1801 Low: 30 mg/kg BS1801 treatment; BS1801 Medium: 90 mg/kg BS1801 treatment; BS1801 High: 180 mg/kg BS1801 treatment; Combine: 30 mg/kg TMZ and 90 mg/kg BS1801 treatment. ns, ∗, ∗∗, ∗∗∗ and ∗∗∗∗ indicated that no significance, p < 0.05, p < 0.01, p < 0.001 and p < 0.0001, respectively.

LN229 and U87 cells were subcutaneous injected into armpit of nude mice to establish in vivo models (Fig. 8A). The mice were treated with different concentrations of BS1801 and TMZ. Obviously, TMZ and BS1801 could inhibit the proliferation of glioma cells (Fig. 8B–C and S8A–B). BS1801 high concentration group had maximal inhibition rate compared with all groups in U87 models (Fig. 8D and S8C), while combined group had maximal inhibition rate in LN229 models (Fig. S8D–E). Meanwhile, we observed BS1801 treatment significantly prolonged the time needed for tumors to reach 100 mm^3^ in both models (Fig. S8F–G). Overall, BS1801 showed a dose-dependent inhibitory effect and exhibited strong antitumor effect on tumor bearing mice. Moreover, we found that mice treated with TMZ alone exhibited reduced weights and organ coefficients, whereas no obvious changes were found in BS1801 treatment groups (Fig. 8E, S8H and Table 2, Table 3), indicating the safety of BS1801. We also found apoptosis cells and ER stress status in tumors were significantly elevated after treatment with different concentrations of BS1801, suggesting that BS1801 may induce U87 cell apoptosis through triggering ER stress (Fig. 8F and S8I–K).

**Table 2:** BS1801 safety evaluation in U87-Bearing mice model.

Group	Body weight (g)	Hepatic organ coefficient (%)	Renal organ coefficient (%)	Cardiac organ coefficient (%)	Splenic organ coefficient (%)	Pulmonary organ coefficient (%)	Cerebral organ coefficient (%)
Control	16.24 ± 0.83	7.46 ± 0.58	2.06 ± 0.25	0.66 ± 0.06	2.18 ± 0.87	0.88 ± 0.10	2.00 ± 0.35
TMZ	13.49 ± 0.74∗∗∗∗	8.44 ± 1.05	2.14 ± 0.06	0.61 ± 0.11	1.32 ± 0.48∗	1.02 ± 0.09∗	2.38 ± 0.13∗
BS1801 Low	14.83 ± 1.43	7.94 ± 0.69	2.15 ± 0.23	0.69 ± 0.12	1.88 ± 0.47	1.27 ± 0.19∗∗∗	2.18 ± 0.34
BS1801 Medium	16.21 ± 1.23	7.55 ± 0.80	1.92 ± 0.17	0.84 ± 0.15∗∗	1.69 ± 0.31	1.03 ± 0.14∗	1.65 ± 0.22
BS1801 High	16.24 ± 1.13	7.98 ± 0.60∗∗∗	1.85 ± 0.18	0.76 ± 0.07∗	1.52 ± 0.33	1.13 ± 0.12∗∗∗	1.78 ± 0.28
Combine	13.05 ± 1.23∗∗∗	7.78 ± 0.83∗∗∗	2.02 ± 0.31	0.87 ± 0.15∗∗	0.70 ± 0.24∗∗	1.09 ± 0.12∗∗	1.91 ± 0.22

Data are shown as Mean ± SD value.

∗, ∗∗, ∗∗∗ and ∗∗∗∗ indicated that p < 0.05, p < 0.01, p < 0.001 and p < 0.0001, respectively.

**Table 3:** BS1801 safety evaluation in LN229-Bearing mice model.

Group	Body weight (g)	Hepatic weight (g)	Renal weight (g)	Hepatic organ coefficient (%)	Renal organ coefficient (%)
Control	17.85 ± 1.02	1.33 ± 0.10	0.30 ± 0.02	7.46 ± 0.58	1.71 ± 0.17
TMZ	16.57 ± 1.45	1.39 ± 0.17	0.30 ± 0.05	8.44 ± 1.05	1.84 ± 0.20
BS1801 Low	18.54 ± 2.10	1.48 ± 0.28	0.36 ± 0.05	7.94 ± 0.69	1.95 ± 0.16
BS1801 Medium	17.36 ± 1.41	1.31 ± 0.12	0.32 ± 0.02	7.55 ± 0.80	1.83 ± 0.13
BS1801 High	17.45 ± 0.92	1.39 ± 0.15	0.31 ± 0.03	7.98 ± 0.60	1.76 ± 0.21
Combine	18.56 ± 2.12	1.44 ± 0.17	0.34 ± 0.03	7.78 ± 0.83	1.82 ± 0.12

Data are shown as Mean ± SD value.

**Supplementary Fig. S9:**
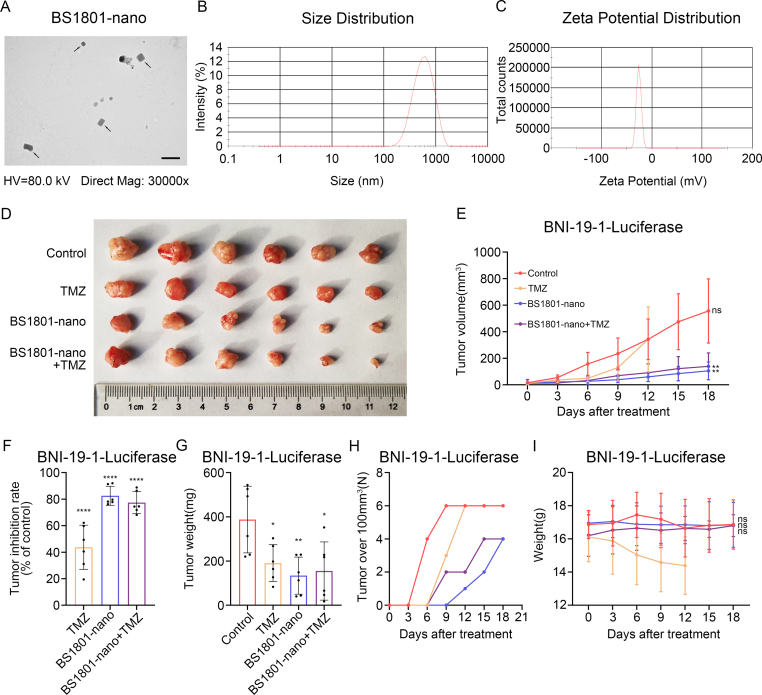
BS1801-nano inhibited glioma proliferation combined with TMZ in vivo. (A) Representative TEM images of BS1801-nano. (B) The size distribution of BS1801-nano. (C) Zeta potential distribution of BS1801-nano. (D) Tumor morphology of BNI-19-1-Luciferase mice models after different drugs treatment. (E) Tumor volume analysis, (F) Tumor inhibition rate analysis, (G) Tumor weight analysis, (H) Tumor growth rate analysis and (I) Body weight analysis of different drug treatments. In subcutaneous BNI-19-1-Luciferase xenograft models (N = 6 for each group): Control: 0.9 % normal saline; TMZ: 30 mg/kg TMZ treatment; BS1801-nano: 90 mg/kg BS1801-nano treatment; BS1801-nano + TMZ: 30 mg/kg TMZ and 90 mg/kg BS1801-nano treatment. ns, ∗, ∗∗ and ∗∗∗∗ indicated that no significance, p < 0.05, p < 0.01 and p < 0.0001, respectively.

To investigate the prospect of BS1801 in clinical application, Blood-Brain-Barrier permeability assay was evaluated by MDCK-MDR1 cells. We found that BS1801 exhibited relatively poor permeability compared to TMZ (Fig. 8G). Therefore, we prepared BS1801 into nanocrystal (BS1801-nano) and the TEM pictures showed that BS1801-nano was in the form of stick (Fig. S9A). We further performed dynamic light scattering (DLS) assay to measure the grain size and zeta potential of BS1801-nano. The results showed that the average grain diameter was 531.8 nm and the zeta potential was −25.9 mV, suggested that BS1801-nano carried negative charges and the solution of BS1801-nano was relative stable (Fig. S9B–C). As expect, the permeability of BS1801-nano was significantly improved (Fig. 8G). Subsequently, we conducted subcutaneous BNI-19-1-luciferase mice model to investigate the efficacy and safety of BS1801-nano. We found that BS1801-nano had favorable tumor inhibition rate and drug safety (Fig. S9D–I and Table 4). Finally, we established intracranial orthotopic BNI-19-1-luciferase mice models (Fig. 8H). We found that the mice in BS1801-nano combined with TMZ group had remarkable inhibition rate and relatively longer survival (Fig. 8I–J). These results revealed that the combination of BS1801-nano and TMZ can significantly inhibit glioma proliferation.

**Table 4:** BS1801-nano inhibition effect and safety evaluation in BNI-19-1-luciferase-bearing mice model.

Group	Sample number (N)	Tumor volume (mm^3^)	Tumor weight (g)	Inhibition rate (%)	Body weight (g)	Hepatic organ coefficient (%)	Renal organ coefficient (%)	Cerebral organ coefficient (%)
Control	6	557.09 ± 241.73	0.39 ± 0.15	–	16.87 ± 1.48	7.88 ± 1.79	1.73 ± 0.29	1.86 ± 0.12
TMZ	6	343.23 ± 245.20	0.19 ± 0.08∗	38.39	14.38 ± 1.73∗	7.12 ± 0.90	1.96 ± 0.29	2.28 ± 0.17∗
BS1801-nano	6	105.18 ± 66.62∗∗	0.13 ± 0.08∗∗	81.12	16.85 ± 1.35	6.48 ± 0.41	1.62 ± 0.11	2.13 ± 0.31∗
BS1801-nano + TMZ	6	139.43 ± 101.18∗∗	0.16 ± 0.13∗	74.97	16.80 ± 0.65	6.45 ± 0.62	1.52 ± 0.15	2.34 ± 0.26∗

Data are shown as Mean ± SD value.

∗ and ∗∗ indicated that p < 0.05 and p < 0.01, respectively.

### 3.8 BS1801 may benefit glioma patients

**Fig. 9:**
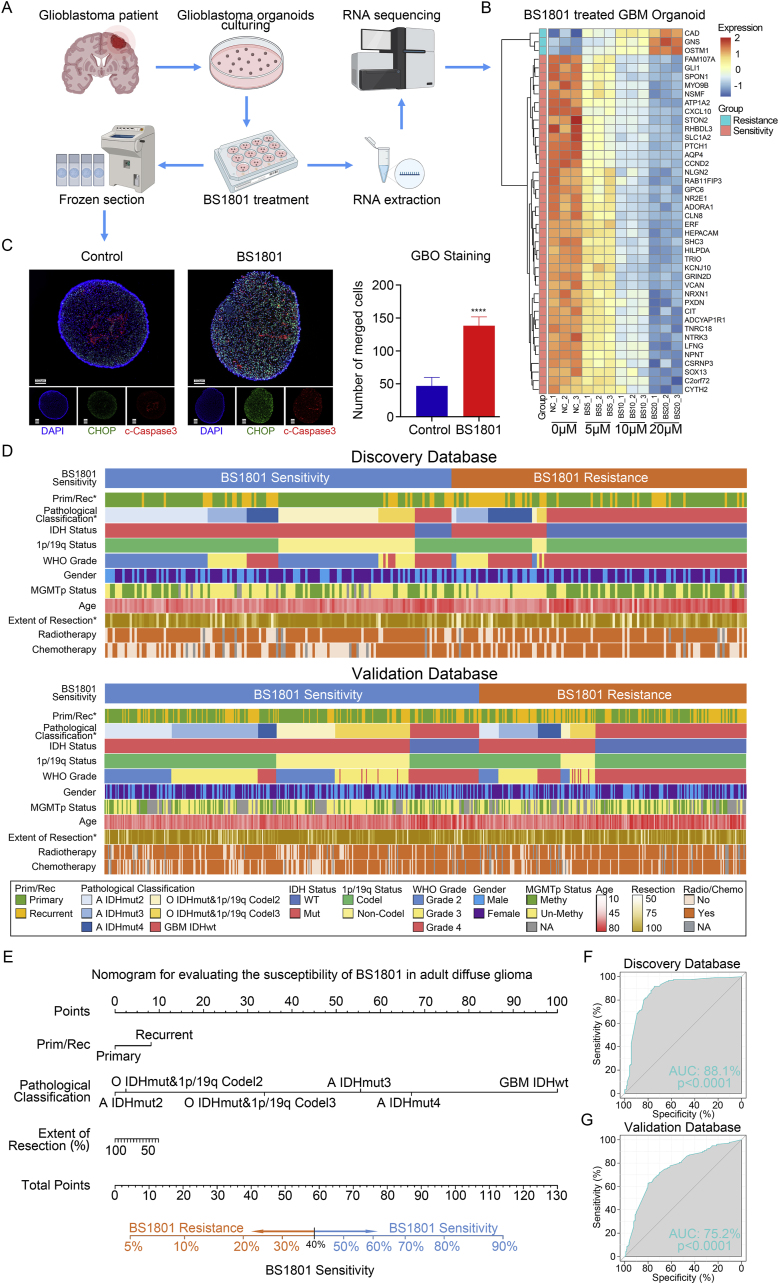
**BS1801 may benefit glioma patients.** (A) The workflow of GBM organoids establishment. (B) Heatmap showed differential gene expression in GBM organoids between control and BS1801 groups with different drug concentrations. (C) Representative IF staining and histogram analysis of CHOP and Cleaved Caspase-3 in GBM organoids after BS1801 treatment. Red fluorescence represented Cleaved Caspase-3. Green fluorescence represented CHOP. Cell nuclei were stained with DAPI. Scale bar was 20 μm. (D) The landscapes of different molecular biomarker status in BS1801 sensitive and resistant group in discovery and validation databases. (E) The nomogram for evaluating the susceptibility of BS1801 in adult diffuse glioma. (F–G) ROC curve analysis showed that the BS1801 sensitive gene list was highly sensitive and specific to predict the susceptibility of BS1801 in discovery and validation databases. ∗∗∗∗ indicated that p < 0.0001.

In the pre-clinical study, we conducted GBM organoids to identify which kinds of glioma patients may be sensitive to BS1801 treatment (Fig. 9A). We performed RNA sequencing for GBM organoids treated with different concentrations of BS1801 and screened out a gene cluster to predict BS1801 sensitivity in patients. (Fig. 9B). Immunofluorescence staining showed that cell apoptosis and ER stress status were upregulated by BS1801 in GBM organoids (Fig. 9C). Subsequently, the landscapes of BS1801 sensitive or resistant glioma patients were displayed in discovery and validation databases, respectively (Fig. 9D). Based on these findings, nomogram was constructed to assess the susceptibility of BS1801 in glioma patients. We found that most of the malignant GBM patients may be sensitive to BS1801. In addition, for relatively low grade glioma patients, recurrent patients with low resection extent may benefit from BS1801 treatment (Fig. 9E). ROC analysis in discovery and validation databases further verified our conclusion (Fig. 9F–G).

## 4 Discussion

Glioma is one of the most common malignant tumors in the central nervous system, characterized by a high recurrence rate [[1], [2], [3], [4],^35^,^36^]. As the only first-line chemotherapeutic drug, TMZ benefits a part of primary glioma patients with MGMT promotor methylation [9,37]. However, residual glioma cells will soon develop TMZ resistance, resulting in tumor recurrence [38]. Unfortunately, patients with recurrent glioma confront a clinical situation where no suitable drug are available [39]. In the present study, through comparing the transcriptome sequencing data from recurrent patients, who relapsed after normative Stupp protocol treatment, and primary patients in our databases, we identified that oxidative stress is a key factor in glioma recurrence. Several studies have demonstrated that oxidative stress was closely associated with tumor proliferation and metabolism [[40], [41], [42], [43], [44]]. Excessive ROS accumulation impairs DNA strand to cause DNA damage [45]. However, our study found that the positive rate of γ-H2AX was significantly decreased in TMZ resistant cells, accompanied by relatively low ROS levels, suggesting that the reduction of oxidative stress levels may contribute to TMZ resistance process.

Through bioinformatics analysis, we identified *TXNRD1* as the most significant upregulated gene in TMZ resistant cells. *TXNRD1* encodes TrxR1, which is a significant component of thioredoxin system [32,[46], [47], [48], [49]]. As an essential anti-oxidant system, thioredoxin system is required for either cancer cells or normal cells to survival [50,51]. Cancer cells usually harbor elevated ROS level due to their uncontrolled proliferation and high metabolism compared to normal cells [52,53]. Therefore, cancer cells may upregulate thioredoxin system to maintain moderate level of ROS and tumor phenotypes, while also rendering cancer cells vulnerable to oxidative stress. Previous studies have described this redox environment as the ‘Achilles’ heel’ of cancer cells [54]. Evidence confirmed that TrxR1 played a crucial role in oxidative stress defense, proliferation, metabolism, metastasis and invasion of cancer cells [48,[55], [56], [57], [58], [59]]. Since organic selenium can bind to Cys497/Sec498 of TrxR1 to inhibit enzyme activity [60], we designed a small-molecular compound BS1801 and investigated its effects. Our study demonstrated that BS1801 treatment significantly elevated ROS levels in glioma cells, resulting in G0/G1 phase arrest, apoptosis and proliferation inhibition.

TrxR inhibitors are classified into natural and synthetic molecules [61]. Natural products are limited in clinical application due to their multiple targets and metabolic instability [32]. Therefore, current clinical trials are almost chemical synthesis TrxR inhibitors. Ebselen revealed extraordinary anti-inflammation effect and was conducted in Phase II clinical trials [62]. Additionally, Ethaselen and Dimesna have shown anti-tumor effect in non-small cell lung cancer [63,64]. Compared with above drugs, BS1801, as a novel TrxR1 inhibitor with two symmetric Se-N covalent bonds, exhibits inhibitory activity against TrxR1 in vitro with a half maximal inhibitory concentration (IC_50_) of 1.03 ± 0.05 μmol/L.

In addition to verifying the therapeutic effect, we also explored the potential mechanism of BS1801. GO analysis showed that upregulating genes after BS1801 treatment were associated with ER stress and UPR. The UPR signal includes three pathways initiated by IRE1α, PERK and ATF6 [[65], [66], [67]]. Our study found that BS1801 triggered ER stress through activating p-IRE1α and p-PERK. Moreover, NAC treatment reversed ER stress, suggesting that ROS may be indispensable for BS1801 to trigger ER stress. We further explored the relevant proteins expression in these two signaling pathways. In IRE1α axis, IRE1α auto-phosphorylated to elicit its RNase activity under ER stress triggered by BS1801. Activated p-IRE1α excised a nucleotide intron from the mRNA encoding transcription factor XBP1. Active XBP1s promoted apoptosis through upregulating DR5 and BAX, with downregulating BCL-2. In PERK axis, the PERK–eIF2α induced ATF4 translation, which can activate the expression of CHOP. Activation of CHOP inhibits cell cycle progress from G1 phase to S phase and promotes apoptosis by modulating BCL-2, BAX and Cleaved Caspase-3 [68]. Unsurprisingly, the combination therapy of BS1801 with IRE1α inhibitor MKC8866 [69] or PERK inhibitor GSK2606414 [70] will rescue ER homeostasis.

Currently, several studies have reported that excessive ROS may induce mitochondrial dysfunction and result in cell apoptosis via mitochondrial depolarization in many cancers [71,72]. Our results revealed that BS1801 treatment dramatically induced mitochondrial depolarization, and these effects were reversed by NAC, indicating that ROS played a crucial role in regulating mitochondrial function. Similarly, BCL-2 and COX IV proteins were downregulated by BS1801 in a dose-dependent manner, further confirming the association between ROS levels and mitochondrial function. Given that COX IV was the vital factor in ATP production, we hypothesized that BS1801 treatment may disturb mitochondrial metabolism. Therefore, mitochondrial stress test was performed to measure OCR following oligomycin, FCCP and Rotenone/Antimycin A treatment. The Seahorse assay data demonstrated that BS1801 treatment significantly decreased basal respiration, max respiration and ATP production, which were indictors to reflect mitochondrial function, indicating that normal mitochondrial metabolic function was impaired. Furthermore, NAC treatment partially reversed this effect, suggesting that BS1801 induces mitochondrial dysfunction by elevating ROS levels.

Our study further found that BS1801 and TMZ exhibited synergistic effect on glioma cells both in vitro and in vivo. The combination group induced higher levels of DNA damage and ER stress compared with TMZ or control group. In glioma-bearing mice models, BS1801 alone or combined with TMZ treatment significantly reduced tumor volume. Additionally, unlike obvious body weight loss in TMZ treatment, BS1801 treatment showed inconspicuous toxicity. To elevate the permeability of BBB, we improved the BS1801 formulation by utilizing the enhanced permeability and retention (EPR) effect of nano-drugs [73]. We found that BS1801-nano can accumulate in tumor tissue, reduce tumor volume and prolong survival combined with TMZ.

In pre-clinical studies, GBM organoids represent the optimal biological models to reflect the therapeutic effect of glioma. We analyzed the RNA-seq data of BS1801-treated GBM organoids in order to screen out patients who may benefit from BS1801 treatment. Interestingly, the results suggested that recurrent GBM patients may be sensitive to BS1801. Meanwhile, based on the safety data of BS1801 in a Phase Ia clinical trial (CTR20210706) involving healthy volunteers, we are conducting a Phase Ib clinical trial (CTR20234234) to further evaluate the safety and efficacy of BS1801 in high grade glioma patients in our hospital.

In conclusion, current treatments remain unable to prevent glioma recurrence due to TMZ resistance. Our study found that the TrxR1 targeting drug BS1801 can inhibit glioma cell proliferation, trigger ER stress, impair mitochondrial function, induce apoptosis and relief TMZ resistance through elevating ROS. It exhibits promising therapeutic effects both in vitro and in vivo. Therefore, BS1801 is a relatively safe drug with potential clinical application in the treatment of recurrent glioma patients.

## CRediT authorship contribution statement

**Yuanhao Chang:** Writing – original draft, Visualization, Investigation, Formal analysis, Data curation, Conceptualization. **Jin Chang:** Validation, Methodology. **Jiayu Ji:** Visualization, Investigation. **Jing Sun:** Investigation, Data curation. **Huimin Hu:** Validation, Methodology. **Bo Pang:** Methodology, Investigation. **Qi Zhang:** Visualization, Software. **Hanwei Yin:** Software, Methodology. **Huihui Zeng:** Formal analysis, Data curation. **Tao Jiang:** Supervision, Resources, Project administration, Funding acquisition. **Guanzhang Li:** Validation, Resources, Funding acquisition, Formal analysis. **Fan Zeng:** Writing – review & editing, Validation, Supervision, Project administration, Funding acquisition, Conceptualization.

## Data availability statement

The datasets analyzed in this study are available from the corresponding authors on reasonable request.

## Declaration of competing interest

The authors declare that they have no known competing financial interests or personal relationships that could have appeared to influence the work reported in this paper.
