# Supplementary material for: Novel Thioredoxin reductase 1 inhibitor BS1801 relieves treatment resistance and triggers endoplasmic reticulum stress by elevating reactive oxygen species in glioma
Source: Redox Biol. 2025 Aug 29;86:103827. doi: 10.1016/j.redox.2025.103827 (PMC12446627; doi:10.1016/j.redox.2025.103827)
Supplement: Multimedia component 4 [file mmc4.docx]

| Antibody | Company | Cat No. | Dilution ratios |
| --- | --- | --- | --- |
| TXNRD1 | Abcam | ab124954 | WB 1:2000 |
| CDK4 | Abcam | ab199728 | WB 1:2000 |
| CDK6 | Abcam | ab151247 | WB 1:1000 |
| Cyclin D1 | Abcam | ab134175 | WB 1:5000 |
| IRE1α | CST | 3294S | WB 1:1000 |
| p-IRE1α | Abcam | ab243665 | WB 1:1000 |
| ATF6 | Abcam | ab227830 | WB 1:1000 |
| PERK | Abcam | ab229912 | WB 1:1000 |
| p-PERK | CST | 3179S | WB 1:1000 |
| eIF2α | CST | 9722S | WB 1:1000 |
| p-eIF2α | CST | 9721S | WB 1:1000 |
| XBP1s | CST | 40435S | WB 1:1000 |
| DR5 | CST | 69400S | WB 1:1000 |
| ATF4 | CST | 11815S | WB 1:1000 |
| BCL-2 | Proteintech | 12789-1-AP | WB 1:1000 |
| BAX | CST | 89400S | WB 1:1000 |
| Cleaved Caspase-3 | CST | 9661S | WB 1:1000; IF 1:200 |
| CHOP | CST | 2895S | WB 1:1000; IF 1:200; IHC 1:50 |
| γ-H2AX | Abcam | ab22551 | IF 1:500 |
| COX IV | Abcam | ab33985 | WB 1:1000 |
| GAPDH | Proteintech | 60004-1-Ig | WB 1:10000 |
| β-actin | CST | 8457S | WB 1:10000 |
| DyLight 488 goat anti-rabbit | Abcam | ab96899 | IF 1:200 |
| DyLight 594 goat anti-rabbit | Abcam | ab96881 | IF 1:200 |

**Supplementary Table S3. Details of Antibodies**
